# Ethnicity-Dependent and -Independent Heterogeneity in Healthy Normal Breast Hierarchy Impacts Tumor Characterization

**DOI:** 10.1038/srep13526

**Published:** 2015-08-27

**Authors:** Harikrishna Nakshatri, Manjushree Anjanappa, Poornima Bhat-Nakshatri

**Affiliations:** 1 Department of Surgery, Indiana University School of Medicine, Indianapolis, IN 46202, USA; 2 Biochemistry and Molecular Biology, Indiana University School of Medicine, Indianapolis, IN 46202, USA; 3 Center for Computational Biology and Bioinformatics, Indiana University School of Medicine, Indianapolis, IN 46202, USA; 4 VA Roudebush Medical Center, Indianapolis, IN 46202, USA

## Abstract

Recent reports of widespread genetic variation affecting regulation of gene expression raise the possibility of significant inter-individual differences in stem-progenitor-mature cell hierarchy in adult organs. This has not been explored because of paucity of methods to quantitatively assess subpopulation of normal epithelial cells on individual basis. We report the remarkable inter-individual differences in differentiation capabilities as documented by phenotypic heterogeneity in stem-progenitor-mature cell hierarchy of the normal breast. Ethnicity and genetic predisposition are partly responsible for this heterogeneity, evidenced by the finding that CD44+/CD24- and PROCR+/EpCAM- multi-potent stem cells were elevated significantly in African American women compared with Caucasians. ALDEFLUOR+ luminal stem/progenitor cells were lower in BRCA1-mutation carriers compared with cells from healthy donors (p = 0.0014). Moreover, tumor and adjoining-normal breast cells of the same patients showed distinct CD49f+/EpCAM+ progenitor, CD271+/EpCAM- basal, and ALDEFLUOR+ cell profiles. These inter-individual differences in the rate of differentiation in the normal breast may contribute to a substantial proportion of transcriptome, epigenome, and signaling pathway alterations and consequently has the potential to spuriously magnify the extent of documented tumor-specific gene expression. Therefore, comparative analysis of phenotypically defined subpopulations of normal and tumor cells on an individual basis may be required to identify cancer-specific aberrations.

Sequencing-based strategies have enabled better characterization of tumor heterogeneity, particularly in breast cancer^1^,^2^. However, there have been few attempts to document heterogeneity in normal breast tissue with respect to proportion of stem, progenitor and mature cells at a given time and potential impact of this heterogeneity on tumor characterization, particularly for transcriptome analysis. Inter-Individual heterogeneity in normal breast tissue due to different rate of differentiation is expected based on recent demonstrations that widespread functional variation in transcriptomes between individuals and individual genotypes could affect the phenotype of normal cells^3^,^4^. Standard approaches such as *in situ* analyses and/or microdissection of different epithelial subpopulations and counting number of terminal duct lobular units have been used to document heterogeneity in the normal breast^5^. Recent studies, using low-throughput and semi-quantitative immunohistochemistry methods, have identified 11 previously undefined cell types in the normal breast based on the expression pattern of the estrogen receptor (ER), the androgen receptor, and the vitamin D receptor^6^. Pregnancy-associated changes in specific cell populations and the related risk of developing breast cancer have been investigated using similar methods^7^.

Heterogeneity in normal breast can have an influence on cancer stem cell (CSC) characterization. The CSC composition of tumors is often determined using cell surface markers such as CD44, CD24, CD271, PROCR (CD201), and DNER, or by intracellular staining for markers such as aldehyde dehydrogenase using the ALDEFLUOR assay^8^,^9^,^10^. CD44+/CD24- and ALDEFLUOR+ cells are the most commonly used markers of breast CSCs^11^,^12^. Basal/triple-negative breast cancers (TNBCs) show enrichment of CD44+/CD24- CSCs, whereas luminal breast cancers are enriched for ALDEFLUOR+ CSCs^13^,^14^,^15^,^16^. All of these CSC markers are expressed in normal breast epithelial cells and inter-individual variability in the number of normal cells expressing CSC markers would make it difficult to claim CSC enrichment in a tumor without characterizing normal cells on an individual basis.

The goals of this study were to document heterogeneity/similarity in profiles of healthy breast tissues, with additional consideration given to ethnicity and genetic predisposition and between tumor and tumor-adjacent normal tissue on an individual basis. This process was accomplished by growing cells from >60 primary samples using epithelial reprogramming assay and combinations of nine markers, which allowed quantitation of at least 20 cell types on an individual basis^17^. We used core biopsies of healthy breast tissue donated to Komen Tissue Bank as a source of normal breast because of documented aberrant histologic characteristics in >85% of breast tissues obtained from reduction mammoplasty or tumor-adjacent normal tissues^18^. The growth conditions used allowed propagation of stem, progenitor and mature cells and the percentage of stem/progenitor/mature cells varied between individuals. We also identified two subpopulations of cells that are enriched in women of African American (AA) ancestry and specific defects in cells from BRCA1-mutant carriers. Comparative analysis of tumor and normal tissue on an individual basis revealed that tumor and adjacent normal cells are phenotypically different in the majority of cases. Thus, although not perfect because epithelial cells are out of their natural environment, the comparison of cells from tumors with the healthy tissue of the contralateral breast or from the adjacent normal of the same individual along with healthy tissue of unrelated donors may be necessary to discern cancer-specific signaling pathway alterations.

## Results

Cells propagated from ~60 primary breast tissues (25 healthy donors, four BRCA1-mutants, three BRCA2-mutants, one hypertrophy, one high-risk, nine tumors with seven adjacent normal tissues from the same patients, two different tumors in two breasts of the same patient, and different pre-cancerous lesions in two breasts of the same patient) were analyzed. These comprehensive analyses documented enormous phenotypic heterogeneity in the normal breast, which may be partially influenced by ethnicity. Consequently, defining ‘global normal’ for comparative analyses with tumor is extremely difficult.

### Phenotypic heterogeneity in the breast epithelial cells of healthy donors

**Figure 1:**
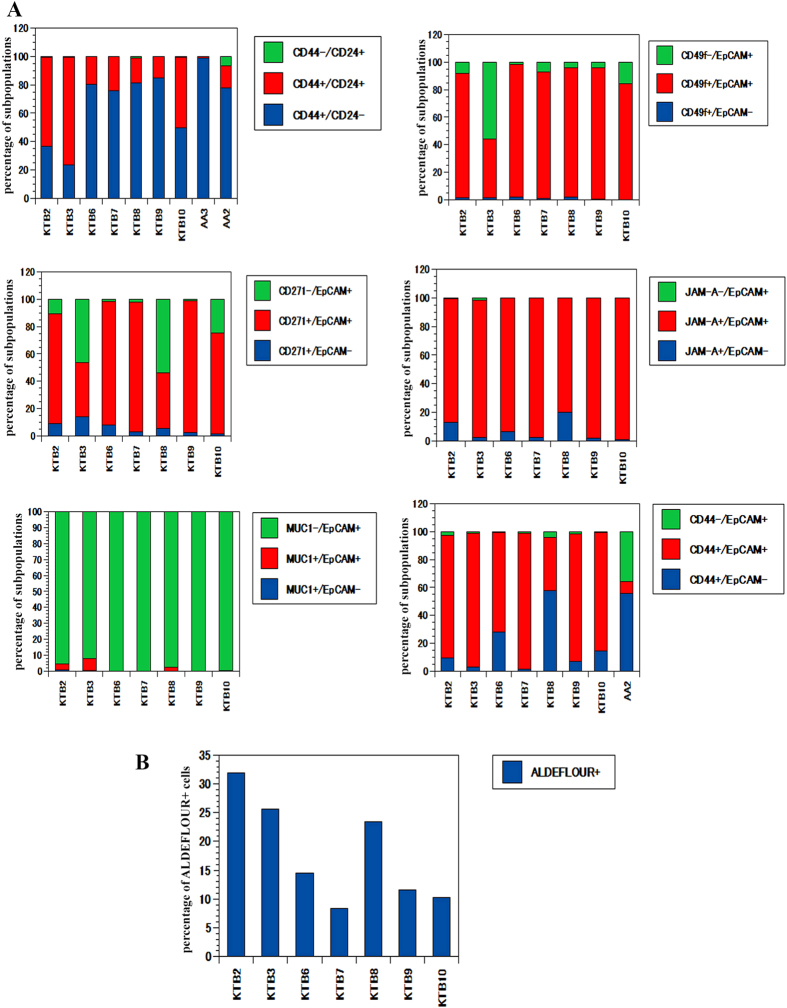
Breast epithelial cells from healthy donors show inter-individual heterogeneity. (**A**) Cells from seven donors were stained with the indicated antibodies, and flow cytometry was used to identify cell subpopulations. Percentage of different cell populations is shown. Raw flow cytometry dot-plots are shown in Figure S2. (**B**) Variable number of ALDEFLUOR+ cells among healthy donors.

Established markers of CSCs, as well as recently discovered markers of CSC in glioblastoma, were used to characterize the phenotypic heterogeneity of the cells propagated from core breast biopsies of healthy donors using an epithelial cell reprogramming assay^17^. Figure 1A provides percentage of different subpopulation of cells present in the breast epithelial cells of seven healthy donors. Additional data on ten other donors can be found in Figure S1. Raw flow cytometry pattern of the cells from these individuals are shown in Figures S2 and S3. These women differed in age, body mass index (BMI) measurements, and prior pregnancies (Table S1). The majority of samples were collected at the luteal phase, and the study included both Caucasian (CA) and African American (AA) women. It is critical to note that because cells showed phenotypic heterogeneity, the assay system was not biased towards the propagation of specific cell subtypes.

#### CD44/CD24 staining pattern

CD44 and CD24 are the most commonly used markers to demonstrate enrichment of CSCs in tumors of specific breast cancer subtypes^9^,^16^,^19^,^20^. We noted remarkable inter-individual variability in the ratio between CD44+/CD24- and CD44+/CD24+ normal cells, possibly reflecting inherent inter-individual differences in the rate of differentiation, irrespective of whether samples were collected at luteal or follicular phase (Fig. 1A).

#### CD49f and EpCAM staining pattern

CD49f^high^/EpCAM^low^, CD49f^high^/EpCAM^medium^, and CD49f^low^/EpCAM^high^ cells are considered to be human breast stem (or basal progenitor), luminal progenitor, and mature/differentiated cells, respectively^21^,^22^. Progenitor and mature populations varied significantly between individuals again highlighting differences in the rate of differentiation (Fig. 1A and Figure S1). Because previous studies have demonstrated significant differences in gene expression between stem, progenitor, and mature cells^21^,^23^, inter-individual variation in the ratio between these three cell types could potentially interfere with the ability to define global “normal” gene expression.

#### CD271 and EpCAM staining pattern

CD271+ tumor cells are basal cells with CSC activity and represent a minor portion of cells in luminal tumors^24^. The number of CD271+/EpCAM-, CD271+/EpCAM+, and CD271-/EpCAM+ cells varied significantly between individuals (Fig. 1A and Figure S1). At present, the biological differences between CD271+/EpCAM- and CD271+/EpCAM+ cells are not known, although distinct gene expression differences between the CD271+/EpCAM- and other epithelial cells have been reported^24^.

#### Jam-A/CD321 and EpCAM staining pattern

The cell adhesion molecule Jam-A/CD321 is expressed in the CSCs of glioblastoma but not in the normal brain^25^. This observation prompted us to evaluate whether Jam-A serves as a unique CSC marker in the breast. Unlike in glioblastoma, Jam-A is expressed in normal breast, and the staining pattern showed remarkable inter-individual variation. In particular, a distinct population of EpCAM^high^/Jam-A+ cells was present in ~50% normal breast tissues (Figures S2 and S3).

#### MUC1 and EpCAM staining pattern

The expression of MUC-1 protein is deregulated in breast cancer and is involved in self-renewal^26^. However, only a few cells were MUC-1+/EpCAM+, and they did not show major inter-individual differences. Thus, not all markers showed significant inter-individual heterogeneity.

#### CD44 and EpCAM staining pattern

CD44 and EpCAM markers have recently been used to distinguish luminal and basal cells^27^. CD44+/EpCAM- cells are basal cells, whereas CD44-/EpCAM+ or CD44+/EpCAM+ cells are luminal cells. Both populations of cells varied significantly between individuals (Fig. 1A and Figure S1).

#### ALDEFLUOR+ staining pattern

Aldehyde dehydrogenase (ALDH)-expressing cells are considered breast CSCs, which are often enriched in luminal/HER2+ breast cancers^12^,^15^,^16^. In the normal breast, ER+/ALDH+ progenitor cells are suggested to originate from ER-/ALDH+ stem/progenitor cells, and the ALDH1a1 isoform is functionally involved in this lineage specification^28^. There was considerable inter-individual variation in the number of ALDH+ cells (as measured using the ALDEFLUOR assay), suggesting that the luminal to basal cell ratio as well as the precursors for ER+ cells vary between individuals (Fig. 1B, S1 and S4).

Because culturing required growth on irradiated murine embryonic fibroblasts as a feeder layer, we ensured that none of these markers was expressed on these fibroblasts (Figure S5A). These cells stained weakly for CD49f but not any other markers. We also stained epithelial cells from few samples for CD45, CD31, and CD140b, markers of hematopoietic, endothelial, and fibroblast cells, respectively (Figure S5B). Less that four percent of cells stained positive for CD45 or CD31. Presence of these small number of contaminating lineage positive cells did not influence our interpretation (Figure S5B). Only epithelial cells stained positive with a pan-cytokeratin antibody, suggesting that the reprogramming assay promotes growth of epithelial cells (representative data are shown in Figure S5C).

### CD44^high^/CD24- cells of AA women express higher levels of genes that regulate stemness, EMT, and the extracellular matrix

**Figure 2:**
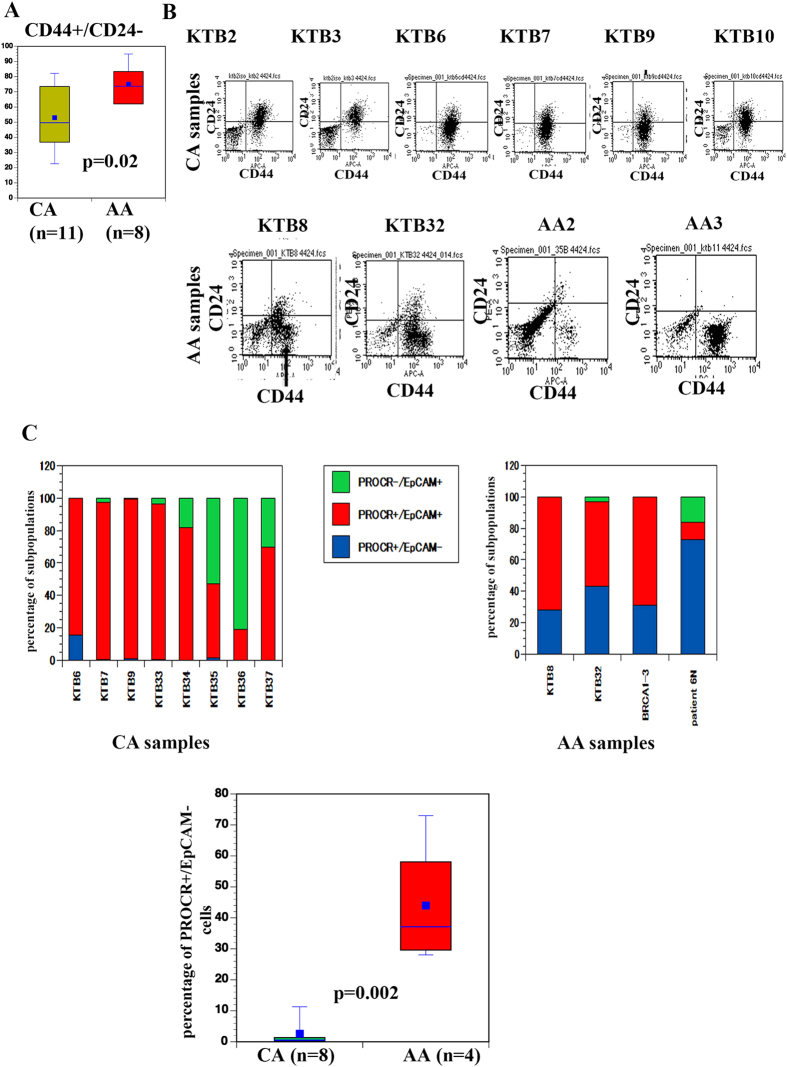
Cells derived from AA-women contain unique CD44^high^/CD24- cells and are enriched for PROCR+/EpCAM-cells. (**A**) Cells derived from AA-women are enriched for CD44+/CD24- cells compared with CA women. (**B**) Cells from AA-women stained for CD44 and CD24 show a distinct CD44^high^/CD24- cells. Pattern of staining from five CA-women and four AA-women are shown. (**C**) PROCR/EpCAM staining pattern of cells from CA-women and AA-women. The top panel shows PROCR/EpCAM staining pattern whereas the bottom panel shows statistical difference in PROCR+/EpCAM- cells between CA-women and AA-women.

Among various marker combinations we examined, CD44/CD24 staining pattern appeared distinct between CA and AA. First, overall number of CD44+/CD24- cells was significantly higher in AA compared with CA (Fig. 2A). Second, cells from several AA donors but not CA donors contained a distinct subpopulation of CD44^high^/CD24- cells (Fig. 2B and Figures S2 and S3). These CD44^high^/CD24- cells are distinct from the CD44+/CD24- cells present in all other samples based on CD44 expression levels. Thus, ethnicity does appear to have quantitative effect on CD44 expression.

CD44^high^/CD24- cancer cells generally overexpress genes associated with stemness and epithelial to mesenchymal transition (EMT)^13^,^29^. To determine whether AA-specific CD44^high^/CD24- cells naturally express higher levels of these genes, we sorted CD44^high^/CD24- and CD44+/CD24+ cells from KTB8 in three biological replicates and subjected them to qRT-PCR of 84 genes that regulate stemness, cell adhesion/invasion, and EMT. The significantly differentially expressed genes between these two subpopulations are shown in Table 1, and the list of genes analyzed with the raw data is in supplementary Table S2. We found specific upregulation of select collagens, CTNBB1, FOXC2, and ZEB1 in CD44^high^/CD24- cells. Ingenuity pathway analyses revealed that genes elevated in the CD44^high^/CD24- cells are part of the TGFβ/Wnt CTNBB1/NF-κB pathway, whereas genes down-regulated in the CD44 ^high^/CD24- cells are the part of the p53 and ER pathway (Figure S6). Thus, CD44^high^/CD24- cells in AA samples may have a unique biology.

We found significant overlap between genes differentially expressed in CD44^high^/CD24- cells and the recently described PROCR+/EpCAM- multi-potent mammary stem cells^30^. This similarity prompted us to determine whether cells from AA-women and CA-women differ in the number of PROCR+/EpCAM- cells. Indeed, cells from AA-women that contained CD44^high^/CD24- subpopulation also showed significantly higher numbers of PROCR+/EpCAM- cells compared with cells from CA-women (n = 7 for CA and 4 for AA, p = 0.0002; Fig. 2C and Figure S7). These results suggest that AA and CA women differ in their breast epithelial cell hierarchy. However, cells prepared from few cryopreserved tissues had lower levels of PROCR+/EpCAM- cells, possibly reflecting sensitivity of these cells to cryopreservation.

### BRCA1-mutant carriers contain a lower number of ADLEFLUOR+ breast epithelial cells

**Figure 3:**
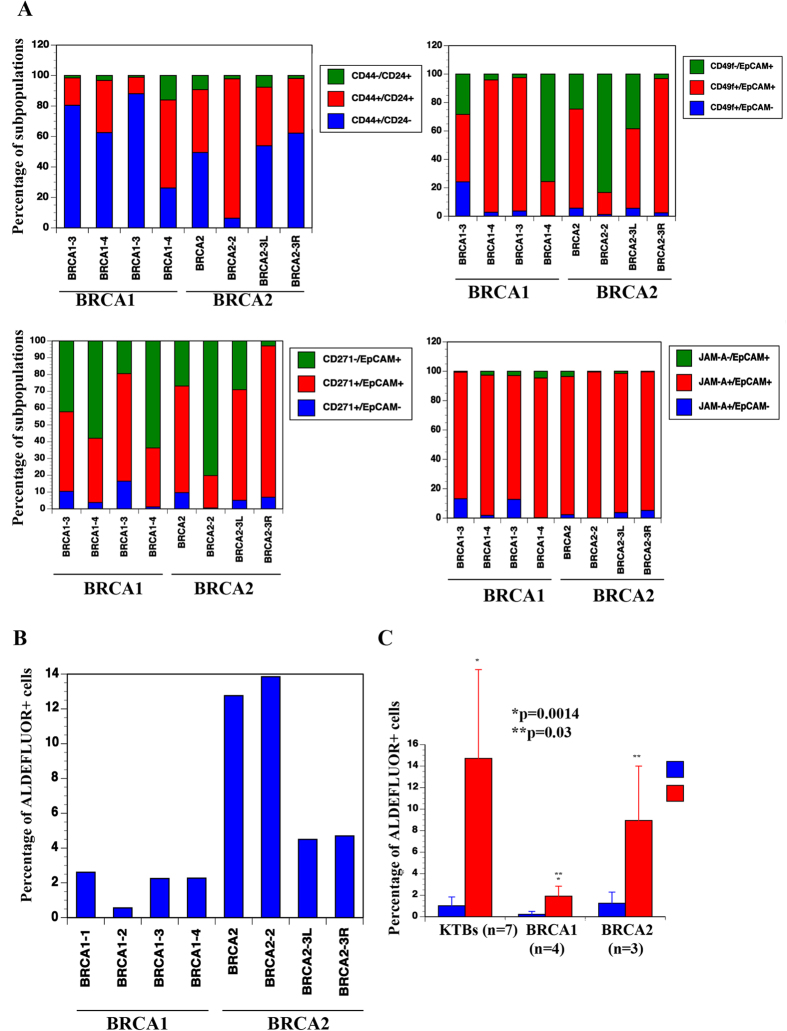
BRCA1-mutant carriers have lower ALDEFLUOR+ cells. (**A**) Breast epithelial cells from four BRCA1-mutant carriers and three BRCA2-mutant carriers were stained with antibodies against the indicated cell surface markers and subjected to flow cytometry. Inter-individual variation in staining pattern is shown. (**B**) BRCA1-mutant carriers have lower numbers of ALDEFLUOR+ cells compared with cells from healthy normal or BRCA2-mutant carriers.

To determine whether the inter-individual variations noted above are linked to the risk of developing breast cancer, we next generated cells from prophylactic mastectomy tissues from various risk groups including four BRCA1-mutant carriers and three cases of BRCA2-mutant carriers. In one of the BRCA2-mutant carriers, cells from two breasts were independently propagated. Interestingly, there were some differences in the levels of differentiated cells between two breasts of the same individual based on CD49f/EpCAM staining pattern (Fig. 3A). However, additional samples need to be tested to determine whether there are regional differences for undifferentiated and differentiated cells within the breast.

Cells from four BRCA1-mutant carriers showed individual variability and did not display unique features compared with healthy donors or other cells from other high-risk patients (Fig. 3A, S8 and S9). Previous studies have demonstrated defects in progenitor lineage commitment in BRCA1-mutant carriers and enrichment of CD49f+/EpCAM- basal cells due to the stabilization of the Slug protein^31^. We did not observe enrichment of CD49f+/EpCAM- cells in any of the BRCA1-mutant cases compared with healthy donors. In fact, cells from the BRCA1 mutant-4 sample were predominantly CD49f-/EpCAM+ and CD271-/EpCAM+ . Our observation is similar to that in a recent publication that failed to observe BRCA1-mutant-specific enrichment of CD49f+/EpCAM- early basal cells compared with healthy donors^32^. A specific BRCA1 mutation is less likely to be responsible for this discrepancy because Pathania *et al.* evaluated cells from 14 types of BRCA1 mutations and did not observe differences in CD49f, CD44 and EpCAM expression profiles^32^. We noted one major difference between BRCA1-mutant carriers and healthy donors; all four BRCA1-mutant carriers contained extremely low levels of ALDEFLUOR+ cells compared with the seven healthy donors or BRCA2-mutant carriers (p = 0.0014 healthy *vs.* BRCA1-mutants; p = 0.03 BRCA1-mutants *vs.* BRCA2-mutants, Fig. 3B,C and Figure S8). Thus, BRCA1-mutant carriers are likely deficient in precursors for ER+ luminal cells, which could explain the higher incidence of TNBCs in BRCA1-mutant carriers.

The BRCA2-mutant carriers demonstrated remarkable inter-individual variability without any uniqueness (Fig. 3A and S8). Cells from a case of hypertrophy, patient with prior history of breast cancer or fibrosis did not display any unique features (Figure S9).

In summation, these results suggest that breast cells from patients at high-risk of developing breast cancer display remarkable phenotypic heterogeneity, which is similar to the level of heterogeneity observed in the general population. The only exception was lower levels of ALDEFLUOR+ cells in the BRCA1-mutant carriers.

### CD49f/EpCAM staining identified phenotypically distinct tumor and adjacent normal cells of the same patient

**Figure 4:**
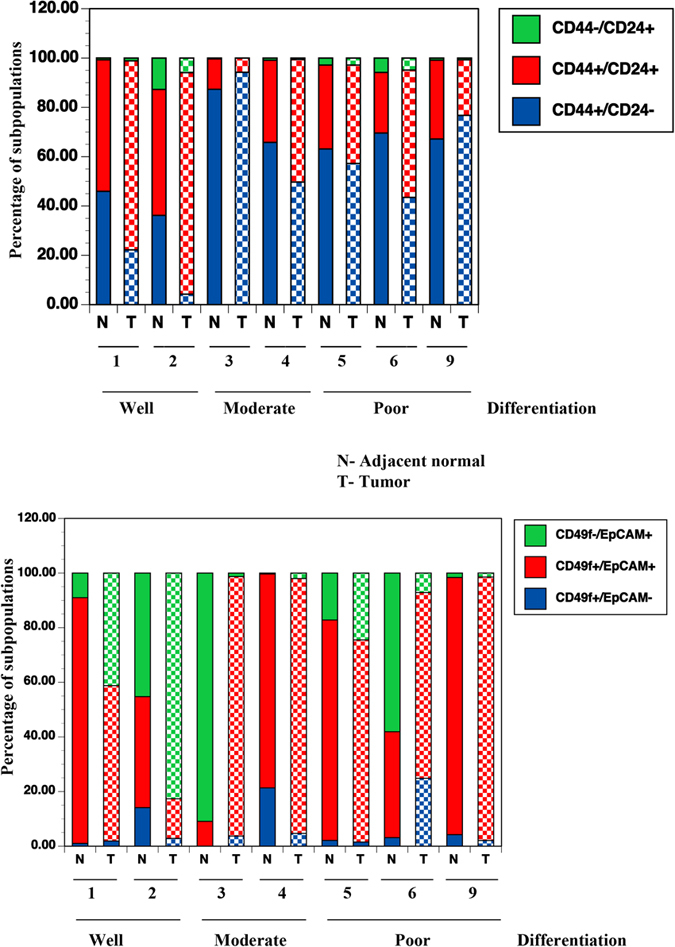
Tumor and adjacent normal cells show differences in CD49f and EpCAM staining pattern. Tumor and adjacent normal cells from seven patients were stained with isotype control, CD44-APC/CD24-PE or CD49f-APC/EpCAM-PE and subjected to flow cytometry. The tumor characteristics, age, and ethnicity of the patients are shown Table S3. CD44+/CD24- cells in tumors are suggested to be CSCs. CD49f+/EpCAM-, CD49f+/EpCAM+, and CD49f-/EpCAM+ cells are considered to represent stem, luminal progenitor, and mature/differentiated cells, respectively. Differentiated tumors contained elevated number of CD49f-/EpCAM+ cells, whereas poorly differentiated tumors contained elevated number of CD49f+/EpCAM+ cells. N = normal; T = tumor.

Having established individual variation in the normal breast, we next examined which of the currently used CSC markers can distinguish tumor from adjacent normal cells and can be used to document tumor-specific enrichment of CSCs. We also used this assay to determine whether the tumor cell phenotype correlated with clinical parameters. The adjacent normal tissues were from mastectomy cases and from the affected breast as distant away as possible from the tumor without compromising specimen integrity; all tumors except one HER2+ tumor and one TNBC were from treatment-naïve cases. Note that the DNA from a limited number of tumor cells was subjected to copy number variation analysis using the NanoString Technology nCounter Cancer CNV v2 code set to confirm genomic aberration in tumor cells. Figure 4 shows quantitative differences in different subpopulation of cells between tumor and adjacent normal of seven patients and raw flow cytometry dot-plots are shown in Figure S10. Age, ethnicity, and tumor characteristics are shown in Table S3. As with normal breast epithelial cells from healthy donors, tumor-adjacent normal showed remarkable heterogeneity. Pair-wise comparison also showed different marker profiles of tumor and adjacent normal ranging from modest differences (patients 4, 5 and 9) to extreme differences (patients 2, 3 and 6) suggesting differences in differentiation status of tumors and adjacent normal. In general, differentiated tumors contained higher levels of CD49f-/EpCAM+ mature cells compared with poorly differentiated tumors.

To further extend the above observations, we characterized tumor cells from two other patients. Patient-7 had an ER+/PR+ moderately differentiated tumor. Tumor cells displayed mature features, as most of the tumor cells were CD49f^low^/EpCAM+, CD271-/EpCAM+, and EpCAM^high^/Jam-A^high^ (Figure S11A). The tumor in patient-8 was a TNBC inflammatory type. Tumor cells were predominantly CD44+/CD24- and CD49f+/EpCAM+ (Figure S11B). Additional features of this tumor such as enriched CD271+/EpCAM^low^ and Jam-A+/EpCAM^low^, cells suggest basal phenotype, which is often manifested by inflammatory breast cancers^33^.

### Tumor and adjacent normal cells show different levels of ALDEFLUOR+ cells

**Figure 5:**
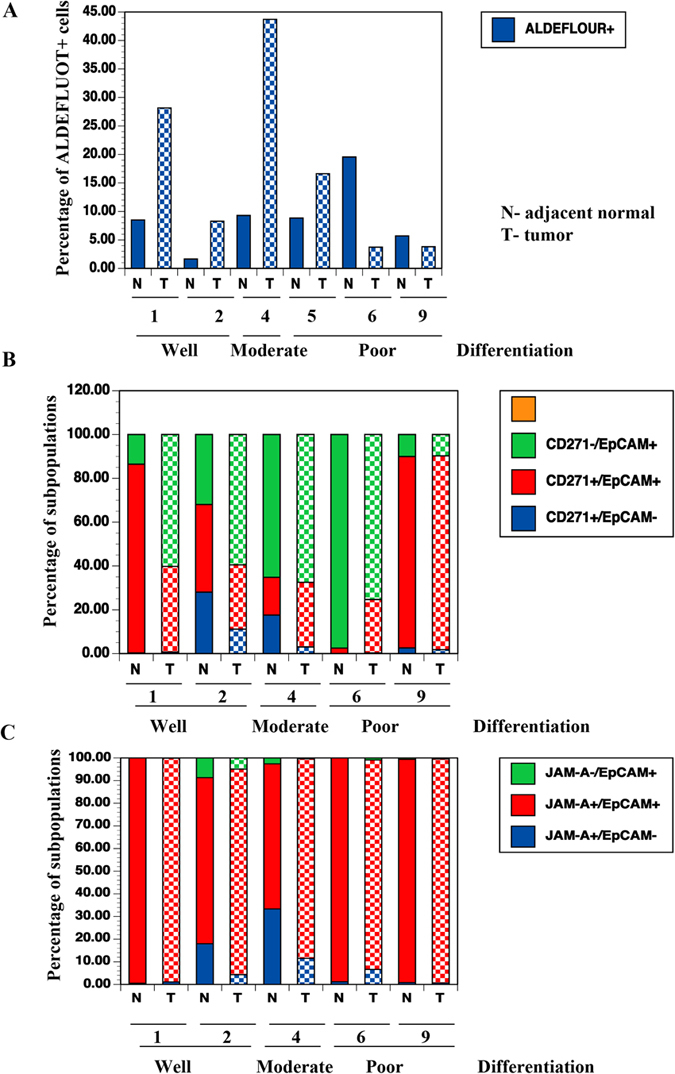
Tumor and adjacent normal cells show differences in ALDEFLUOR+ cells. (**A**) The ALDEFLUOR staining pattern in tumor and adjacent normal cells from six patients. (**B**) CD271-APC/EpCAM-PE staining pattern of tumor and adjacent normal cells. (**C**) Jam-A-PE/EpCAM-APC staining pattern of tumor and adjacent normal cells.

To document additional differences between the tumor and adjacent normal cells, we stained matched normal and tumor cells from five patients with ALDEFLUOR. Based on previous observations^14^,^16^, luminal breast cancers were expected to demonstrate an elevated number of ALDEFLUOR+ cells compared with adjacent normal tissues. However, that did not appear to be the case always. Although the tumor in patient-5 was a TNBC, her tumor was enriched for ALDEFLUOR+ cells compared with adjacent normal tissue (Fig. 5A and Figure S12A). By contrast, the poorly differentiated ER+/PR+ tumor in patient-6 had a lower number of ALDEFLUOR+ cells compared with adjacent normal tissue. Pateint-1 and patient-2 with differentiated tumors and patient-4 with HER2+ tumor had higher levels of ALDEFLUOR+ cells compared with their adjacent normal tissue (Fig. 5A). The ER+/PR+ tumor of patient-7 had higher levels of ALDEFLUOR+ cells compared with the poorly differentiated inflammatory breast cancer of patient-8 (Figure S11). Four out of six tumors displayed higher levels of ALDEFLUOR+ cells compared with adjacent normal tissue.

Similar to ALDFLUOR, we noted differences in the staining pattern of CD271/EpCAM between tumor and adjacent normal with poorly differentiated tumors showing higher levels of CD271+/EpCAM+ cells compared with differentiated tumors (Fig. 5B, Figure S12B). By contrast, Jam-A/EpCAM did not show a marked difference between tumor and adjacent normal tissue with the exception of cells from differentiated tumor cells of patient-1 and patient-2 having a distinct Jam-A^medium^/EpCAM^high^ subpopulation (Fig. 5C and Figure S12C).

In summation, the results presented in Figs 4 and 5 indicate that CD49f/EpCAM, ALDEFLUOR, and CD271/EpCAM staining are better at distinguishing tumor cells from normal cells on an individual basis. Critically, phenotype of cultured tumor cells showed differentiation characteristics similar to the original tumor suggesting the relevance of this culturing method for better characterization of tumors.

### Cancer-specific aberrations are involved in conferring phenotypic diversity to tumor cells

**Figure 6:**
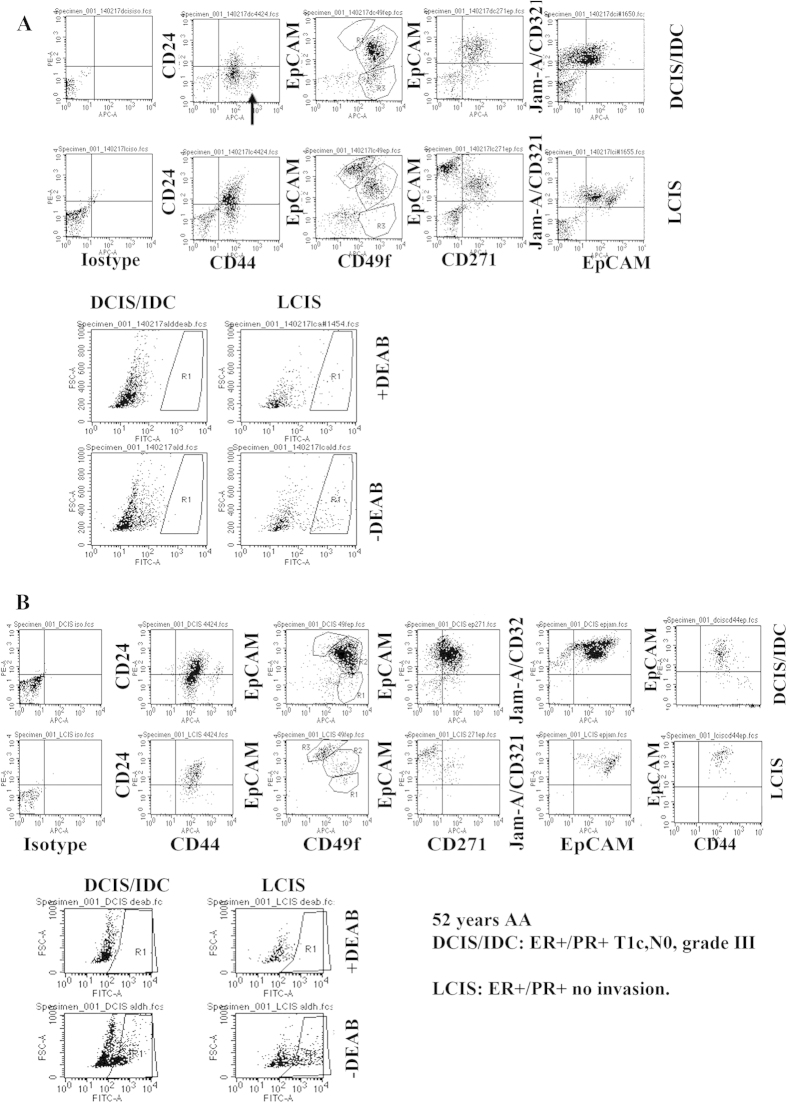
Two tumor types in the same patient show distinct marker profiles. (**A**) DCIS/IDC and LCIS cells from the same patient grown on a feeder layer display distinct marker profiles. These samples were from an AA-woman, and a CD44^high^/CD24- subpopulation of cells is evident in the DCIS/IDC sample. (**B**) Similar assay as above except that cells were adapted to grow in 2D without a feeder layer for two weeks. The 2D condition increased the intensity of the EpCAM staining.

Although marker expression profiles of tumor cells correlated closely with their pathologist-assigned differentiation status, additional proof is needed to show that genomic aberrations are associated with the phenotype of these tumor cells. Towards this goal, we characterized two distinct subtypes of tumors from the same patient. This AA patient had ER+/PR+, node negative multifocal invasive ductal carcinoma associated with DCIS in her right breast (DCIS/IDC) and lobular carcinoma *in situ* (LCIS) in her left breast. Previous studies have already documented different molecular aberrations/genetic predisposition in LCIS versus IDC^34^. DCIS/IDC and LCIS cells showed clear differences in their phenotype. For example, distinct CD44^high^/CD24- and CD44^high^/EpCAM+ subpopulations were present in DCIS/IDC, but not in LCIS (Fig. 6A). As noted above, similar CD44^high^/CD24- cells are present in AA-women. DCIS/IDC samples contained higher levels of CD44+/CD24- cells compared with LCIS. DCIS/IDC cells were predominantly CD49f+/EpCAM+, whereas LCIS had equal levels of CD49f+/EpCAM+ luminal progenitor and CD49f^low^/EpCAM+ mature cells (Fig. 6A). Similar differences were noted with respect to the CD271 and Jam-A staining patterns, all pointing to more differentiated/luminal features of LCIS compared with DCIS/IDC. Consistent with this possibility, the LCIS sample contained a higher percentage of ALDEFLUOR+ cells. The results of another unusual case that involved a right breast with stromal fibrosis with microcalcification, whereas the left breast of the same patient with unusual ductal hyperplasia, microcalcification and fibroadenomatous change, are shown in Figure S13. CD44/24, CD49f/EpCAM, CD44/EpCAM, and ALDEFLUOR staining patterns were different between cells from the two breasts. Thus, cancer-specific aberrations contribute to phenotypic changes in the cancer cells.

Both the normal and tumor samples analyzed above were grown on a feeder layer and with ROCK inhibitor. Therefore, some of the phenotypic features can be attributed to the growth conditions because factors secreted by the feeder layer, or physical contact between epithelial cells and the feeder layer, determine the phenotype of the epithelial cells. To rule out this possibility, we adapted DCIS/IDC and LCIS cells to grow in 2D culture without the feeder layer and ROCK inhibitor and then evaluated cells for phenotypic markers. Tumor cells grown under this growth condition were phenotypically similar to cells grown on a feeder layer, although cells that were “differentiated” (for example, EpCAM^high^/Jam-A^high^ and ALDEFLUOR+ cells) were higher under the 2D culture without the feeder layer (Fig. 6B). Differences between DCIS/IDC and LCIS persisted under the 2D growth conditions. Thus, phenotypes observed with reprogrammed cells are likely intrinsic properties. Reprogramming growth conditions slow the differentiation process and allow for quantification of both undifferentiated/differentiated and basal/luminal cells. This is a marked improvement over previously described growth conditions, which, depending on growth factors in the media, enrich for either luminal or basal cells^35^.

## Discussion

This study establishes phenotypic heterogeneity in the normal breast, which can be partially attributed to ethnicity and genetic susceptibility to breast cancer. We have also documented that tumor and adjacent normal cells from the same patient are in different state of differentiation, which could have an impact on identifying genes that are causally linked to the disease by differential gene expression analysis. We initiated this study to determine the ideal normal breast tissue as a reference because of documented histologic abnormalities in reduction mammoplasty or tumor adjacent tissues used routinely as reference samples^18^. Although tissues from healthy donors are certainly better than reduction mammoplasty samples because there are limited proliferative changes in these tissues, our observation of phenotypic heterogeneity with ethnicity as a confounding factor indicates that selection of normal reference breast tissues is substantially more complicated than previously envisioned. The best option may be to use breast tissues from the contralateral breast for comparative analysis with the tumor on an individual basis. However, this is challenging to apply in a clinical setting in the absence of a proven benefit to patients. For that reason, we performed all of our tumor-normal pair comparison using tissues from complete mastectomy cases, thus allowing us to characterize normal tissue from as far away as possible from the tumor.

### Ethnicity influences the normal breast hierarchy

Normal breast reference in large-scale studies, including TCGA, did not consider ethnicity when selecting tissues^36^. We observed a unique subpopulation of cells in majority of African American women that are characterized by a higher expression of CD44, but lacking CD24 or EpCAM. The difference in this population of cells between AA and CA patients is significant (p = 0.01, N = 11 for CA, none positive and N = 10 for AA, 5 positive, Fisher’s exact test, two tailed). These cells expressed higher levels of genes associated with stemness and EMT, and this expression pattern was markedly similar to genes expressed in PROCR+/EpCAM- mammary stem cells^30^. Interestingly, all samples that had this unique population were enriched for PROCR+/EpCAM- cells. Because basal cells display the CD44+/CD24- or the CD44+/EpCAM- phenotype, and cells with these phenotypes often express higher levels of transcription factors involved in EMT^13^,^14^,^27^, it is possible that women of AA ancestry have differences in the following: the basal to luminal cell ratio; the rate of stem to luminal differentiation; the chromatin organization that permits quick transition between epithelial and mesenchymal states; and consequently a difference in susceptibility to breast cancer. AA-women encounter a higher incidence of TNBCs, which are usually enriched for EMT-associated genes^37^. These findings are interesting in the context of recent observation of elevated Wnt, Aurora Kinase A/B, EZH2 and polo-like kinase signaling networks in TNBCs of AA-women compared with TNBCs of CA-women^38^. It is possible that subpopulation of cells in AA-women naturally have elevated activity of these signaling networks and tumors may have originated from such cells. Functional studies of this subpopulation of cells may reveal the biology behind TNBCs in AA-women. Mechanistically, elevated levels of PROCR+/EpCAM- cells in AA could be related to haplotypes of this gene; there are four haplotypes and H1 among them is associated with increased levels of membrane associated PROCR^39^.

### Phenotypic differences between healthy normal and BRCA1-mutant cells

Several breast cancer susceptibility genes have been identified over the years but phenotypic markers that can distinguish breast epithelial cells of high-risk patients have yet to be identified^40^. Recent studies with limited number of samples showed a defect in luminal progenitor commitment and accumulation of stem cells among BRCA1 mutants, which was mechanistically linked to stabilized EMT-associated Slug protein^31^. However, another study failed to observe elevated Slug in BRCA1 mutant cells^32^. We did observe significantly lower ALDEFLUOR+ cells in all BRCA1-mutant samples. Because ALDEFLUOR+ cells are precursors for ER+ luminal cells^28^, future studies may focus on documenting differences in the differentiation of stem/progenitor cells to mature cells in high-risk patients compared with healthy individuals. In addition, differences in ER+/ALDEFLUOR+ and ER-/ALDEFLUOR+ cells could serve as markers of differentiation defects. Our results differ from other reports, which showed elevated ALDEFLUOR+ cells upon BRCA1 knockdown in human breast cancer or epithelial cell lines and in mouse mammary epithelial cells^41^,^42^,^43^. Reasons for this discrepancy are unknown but could be related to differences in source of cells (cell lines versus primary cells) and experimental conditions (knockdown versus the presence of mutant BRCA1 proteins).

### Impact on studies related to cancer-specific gene expression

In addition to inter-individual variation in breast epithelial cells among healthy donors (Fig. 1 and Figure S1), we observed phenotypically divergent adjoining normal and tumor cells in the same patient. For example, the adjacent normal cells from patient-3 contained CD49f-/EpCAM+ mature cells, whereas the tumor cells were mainly luminal progenitors (Fig. 4). In the case of differentiated tumors (patient-1 and patient-2), the situation was reversed. In the normal breast, more than 2000 genes are differentially expressed between differentiated luminal and bipotent or luminal-restricted progenitor cells^23^. Thus, if adjacent normal cells and tumor of the same patient differ in their differentiation status, most of the gene expression differences between normal and tumor could be the result of variations in differentiation status, and the majority of differentially expressed genes are not causally linked to cancer. This possibility raises concerns about the use of resources such as Oncomine and Ingenuity Pathways in assessing cancer-associated gene expression changes by comparing cancer with a global “normal” instead of individual-specific “normal”. A recent study that utilized single cell RNA-seq further exposed the limitations of current approaches. Although RNA-seq analysis of bulk glioblastoma enabled classification of the tumor into a specific subtype, single cell RNA-seq showed the presence of alternate subtypes in the same tumor^44^. Moving forward, it may be essential to determine gene expression differences and signaling networks in phenotypically similar normal and tumor cells from the same patient.

## Materials and Methods

### Primary cell propagation

All tissues for the study were de-identified, and Indiana University Institutional Review Board considered the protocol as non-human subject research. Tissues were from healthy donors or from mastectomy cases. Informed consent was obtained from all subjects. All experiments were carried out in accordance with the approved guidelines. The Komen Tissue Bank collected healthy normal tissues, whereas Indiana University Simon Cancer Center (IUSCC) Tissue Bank collected tumor and adjacent normal. Standard operating procedure for collecting and processing of normal tissue can be found in the Komen Tissue Bank website. For tumors and adjacent normal, a pathologist grossly examined each case for size of tumor including inking margins, if necessary, prior to any dissection. Normal adjacent or tumor tissue was grossly determined by the pathologist and a representative section from each specimen was submitted for Formalin-fixation, paraffin-embedding (FFPE) and Quality Control (QC). The normal adjacent tissue was obtained from a distant area of the larger specimen not grossly involved with tumor (or margins) and that showed evidence of breast epithelium (i.e. not solely adipose tissue). The tissue remaining after QC sections was snap frozen and/or placed in study defined nutrient media. A H&E stain was then prepared from each of the FFPE samples and reviewed by a pathologist for diagnosis confirmation.

Freshly collected tissues or tissues cryopreserved after mincing and storing in Lonza freezing media (Lonza, Basel, Switzerland) with ROCK inhibitor were dissociated using a collagenase/hyaluronidase mixture (300 μl in 2.7 ml of media, Stem Cell Technologies, Vancouver, Canada) plus ROCK inhibitor (5 μM, Lonza) for two hours at 37 °C. The dissociated cells were filtered through sterile 70-micron filters, pelleted, washed in PBS, and plated on irradiated murine embryonic fibroblasts (Applied StemCell, Inc., San Jose, CA, USA) using the media described previously^17^ with some modifications. To reduce metabolic acidosis, we used a low glucose (1 gm/liter) DMEM and F12 mixture (1:3) and avoided DMSO as a solvent for any reagents to reduce stem cell differentiation^45^. The media additionally contained 5% fetal bovine serum, 5 μg/ml of bovine insulin, 0.4 μg/ml hydrocortisone, and 20 ng/ml EGF and penicillin-streptomycin. Irradiated fibroblasts (~400,000) were plated on 60-mm plates in DMEM plus 10% FBS a day before and washed in PBS before the addition of breast epithelial cells. Tissues that yielded multiple colonies were propagated further to avoid analysis of clonally selected cells. The media were changed every two days and/or the cells were passaged onto new feeder layer every 2-3 days. The cells first were incubated with trypsin at room temperature for 1-2 minutes to remove the feeder layer. After washing in PBS, an additional trypsinization was performed at 37 °C for 5–10 minutes. The trypsinization time varied from sample to sample and was monitored carefully to ensure full trypsinization. Complete trypsinization was necessary for reproducible documentation of the different subtypes of cells. All samples used in the study were of <10 passages and were in culture for <30 days. Several samples were analyzed within the second passage, and cells reanalyzed after additional passages were phenotypically similar to the cells analyzed earlier. The heterogeneity between samples clearly demonstrates that the culture conditions did not favor outgrowth of a specific subpopulation of cells. Cells from nine tumor samples, one adjacent normal and one healthy normal were subjected to nCounter® Cancer CNV v2 evaluation (NanoString Technology) to ensure that tumor cells indeed contained genomic aberration. However, contamination of tumor cells with normal breast epithelial cells cannot be ruled out.

### Flow cytometry analysis

Cells were stained using the indicated antibodies and were analyzed using a BD LSR II flow cytometer, and the data were analyzed using CellQuest or FlowJo software. The antibodies used for the study are listed in Table S4. Forward and Side scatter were used to ensure that only live cells were considered in the analysis. Please note that gating was done using appropriate isotype control antibodies (included for all three fluorochromes- FITC, PE and APC) and only a representative isotype control for two fluorescent markers is shown.

### Human epithelial to mesenchymal transition (EMT) PCR array

CD44^high^/CD24- and CD44+/CD24+ cells from the AA healthy donor (KTB8) were sorted by flow cytometry, and RNA was prepared immediately after sorting using the RNAeasy kit (Qiagen, Valencia, CA, USA). To avoid complexity due to inter-individual heterogeneity in the genome, cells from the same individual at three different passages were sorted, and RNA from biological triplicates was subjected to three independent quantitative reverse transcription-polymerase chain reactions (qRT-PCR) using the PCR Array from Qiagen/SA Biosystems (catalogue #PAHS-090A). The array contained 84 EMT/differentiation- and development-associated genes and five reference genes. All five-reference genes were used for normalization. Table S2 contains the list of genes analyzed, the fold changes between the CD44^high^/CD24- and the CD44+/CD24+ cells and the *p* values calculated using the computational program provided by the manufacturer.

### Statistical analyses

The Graphpad QuickClacs program (Graphpad.com) was used for all statistical analyses. Differences in the CD44^high^/CD24- subpopulation of cells between AA and CA patients were analyzed using the Fisher’s exact test (2 × 2 contingency table), whereas an unpaired *t* test to compare between two means was used to analyze differences in PROCR+/EpCAM- cells between AA-women and CA-women and differences in ALDEFLUOR+ cells between BRCA1-mutant patients and healthy donors. Average ± standard deviation is shown in figures.

## Additional Information

**How to cite this article**: Nakshatri, H. *et al.* Ethnicity-Dependent and -Independent Heterogeneity in Healthy Normal Breast Hierarchy Impacts Tumor Characterization. *Sci. Rep.* **5**, 13526; doi: 10.1038/srep13526 (2015).

## Supplementary Material

Supplementary data

Supplementary Table S2

## Figures and Tables

**Table 1:** Genes elevated or down-regulated in CD44^high^/CD24- cells compared with the CD44+/CD24+ cells of an AA woman.

	Genes elevated in CD44^high^/CD24- cells	Fold change	P value
1	CAMK2N1	2.63	.041
2	COL3A1	13.86	.041
3	COL5A2	10.03	.046
4	CTNNB1	10.27	.012
5	FOXC2	2.63	.035
6	GSC	6.47	.013
7	IL1RN	19.61	.051
8	ITGAV	6.18	.024
9	PDGFRB	13.55	.017
10	SERPINE1	6.03	.0060
11	SPARC	7.43	.033
12	TGFB1	2.69	.0080
13	TWIST1	8.53	.015
14	WNT5A	6.77	.011
15	ZEB1	5.63	.022
Genes down-regulated in CD44^high^/CD24- cells			
1	CAV2	.21	.017
2	CDH2	.14	.018
3	DSP	.13	.015
4	EGFR	.44	.007
5	FGFBP1	.23	.051
6	IGFBP4	.44	.031
7	KRT14	.42	.009
8	KRT19	.07	.025
9	PLEK2	.07	.0015
