# Supplementary material for: Ethnicity-Dependent and -Independent Heterogeneity in Healthy Normal Breast Hierarchy Impacts Tumor Characterization
Source: Sci Rep. 2015 Aug 27;5:13526. doi: 10.1038/srep13526 (PMC4550930; doi:10.1038/srep13526)
Supplement: Supplementary data [file srep13526-s1.pdf]

Ethnicity-Dependent and -Independent Heterogeneity in Healthy Normal Breast Hierarchy Impacts Tumor Characterization

Harikrishna Nakshatri[1,2,3,4*], Manjushree Anjanappa[1], and Poornima Bhat-Nakshatri[1],

Departments of Surgery[1], Biochemistry and Molecular Biology[2], Center for Computational Biology and Bioinformatics[3], Indiana University School of Medicine, Indianapolis, IN 46202, USA; VA Roudebush Medical Center[4], Indianapolis, IN 46202, USA

Supplementary data (except Table S2)

Table S1: Age, BMI, Ethnicity and pregnancy history of healthy donors at the time of tissue collection. CA, Caucasian; AA, African American; NA, not applicable. Unknown menstrual cycle indicates substantial gap from the last period.

Number	Age	Ethnicity	BMI	Pregnancy	Menopausal status	Menstrual cycle at time of collection
KTB2	23	Mixed	37.6	0	Pre	Luteal
KTB3	42	CA	28	0	Pre	Luteal
KTB6	31	CA	29.6	0	Pre	Luteal
KTB7	38	CA	30	2	Pre	Follicular
KTB8	35	AA	34.4	6	Pre	Luteal
KTB9	62	CA	24.8	6	Post	NA
KTB10	41	CA	20.9	2	Pre	Unknown
KTB32	48	AA	21.3	0	Post	NA
KTB33	29	CA	22.6	0	Pre	Follicular
KTB34	37	CA	19.8	2	Pre	Luteal
KTB36	28	CA	29.5	2	Pre	Follicular
KTB37	34	CA	22.7	1	Pre	Luteal
KTB39	33	AA	24.5	1	Pre	Unknown
KTB40	35	AA	23.4	1	Pre	Luteal
KTB41	35	AA	22.4	2	Pre	Luteal
KTB42	39	AA	41.8	1	Pre	Unknown

Table S3: Characteristics of tumors that were grown and analyzed by flow cytometry. Age and ethnicity of patients are also indicated.

Patient #	Histopathology	Age	Ethnicity	Tumor grade	Nodal Status	Tumor grade	ER/PR/Her2 Status	Differentiation
1	IDC	46	Caucasian	T1c	N0		+/+/-	Differentiated
2	IDC	56	African-American	T1a	N0		+/+/-	Differentiated
3	Mixed lobular and ductal carcinoma	45	Hispanic	T2	N2a	III	+/+/-	Moderately differentiated
4*	IDC	44	African-American	T1c	N1a	II/III	+/-/+	Moderately differentiated
5	IDC	70	Caucasian	T2	N1a	III	-/-/-	Poorly differentiated
6	IDC	44	Caucasian	T2	N0	III	+/+/-	Poorly differentiated
7	IDC	40	African-American	T2	Nx	III	+/+/Her2 neg (2+)	Moderately differentiated
8	TNBC inflammatory	54	Caucasian	T4b	N2a		-/-/-	Poorly differentiated
9*	TNBC-IDC	44	African American	T1c	N0	III	-/-/-/	Poorly differentiated

*Received neoadjuvant chemotherapy

Table S4: List of antibodies and other flow cytometry reagents used in the study.

Antibody	Catalogue Number	Source
CD44-APC	559942	BD Pharmingen
CD24-PE	555428	BD Pharmingen
CD49f-APC	FAB13501A	R&D Systems
EpCAM-PE	130-091-253	Miltenyi Biotech
EpCAM-APC	130-091-254	Miltenyi Biotech
Jam-A-PE	552556	BD Pharmingen
MUC-1-FITC	559774	BD Pharmingen
CD271-APC	345108	Biolegend
PROCR (CD201)-PE	130-105-256	Miltenyi Biotech
Pan-Cytokeratin-FITC	F3418	Sigma Aldrich
ALDEFLUOR	01700	Stem Cell Technologies
CD140b-PE	558322	BD Biosciences
CD24-FITC	555427	BD Pharmingen
CD49f-FITC	555735	BD Pharmingen
CD45-PE	130-080-201	Miltenyi Biotech
CD31-PE	130-092-653	Miltenyi Biotech

Figure S1: Breast epithelial cells from healthy donors show inter-individual heterogeneity. Percentage of different subpopulation of cells from self-reported five Caucasian and four African American women is shown.

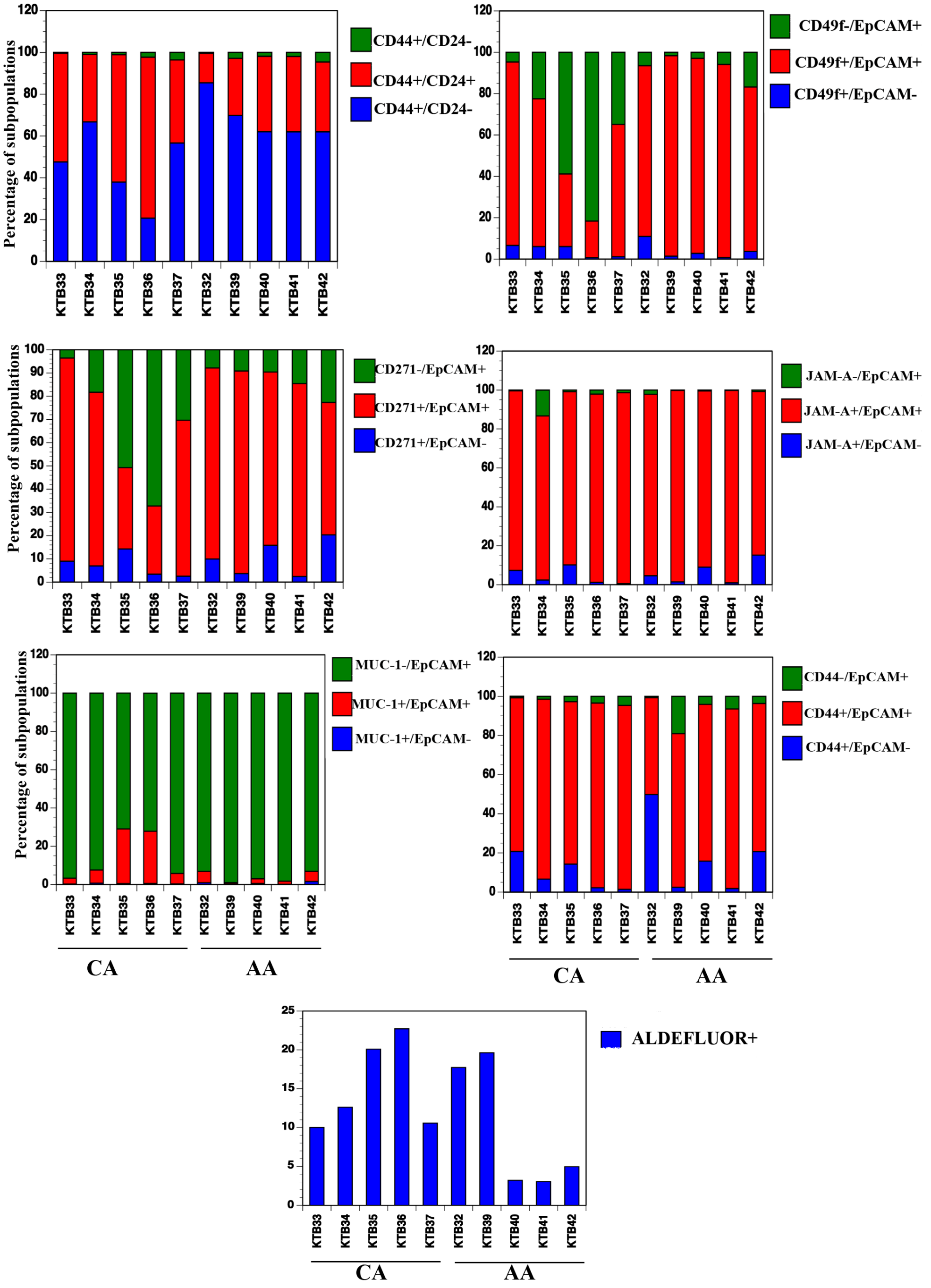

Figure S2: Breast epithelial cells from healthy donors show inter-individual heterogeneity. Cells from seven donors were stained with the indicated antibodies, and flow cytometry was used to identify cell subpopulations. The vertical arrow indicates a unique $CD44^{high}$/CD24- subpopulation in an AA donor. This figure shows raw flow cytometry pattern of data presented in Figure 1

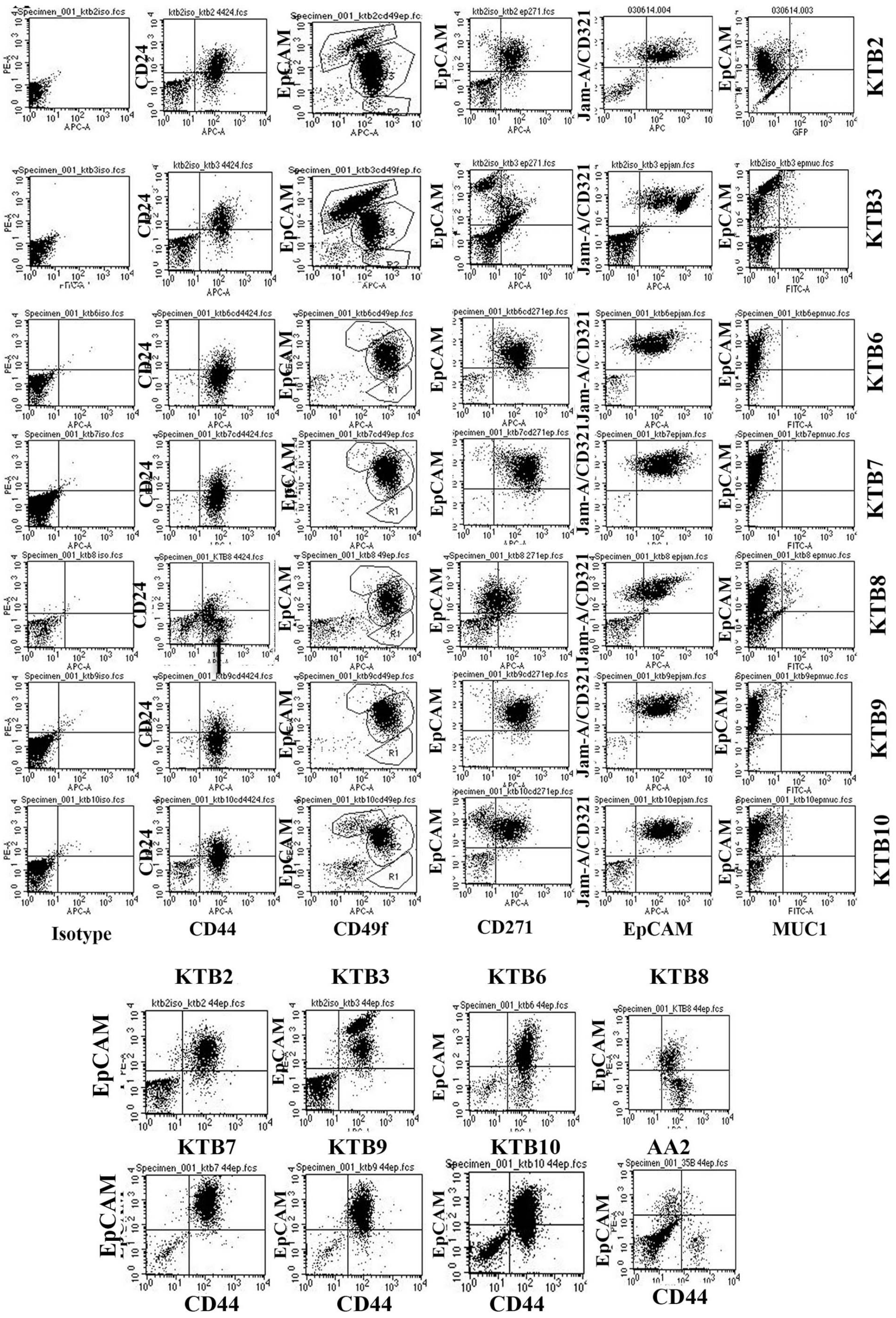

Figure S3: Flow cytometry dot blots of data presented in Figure S1.

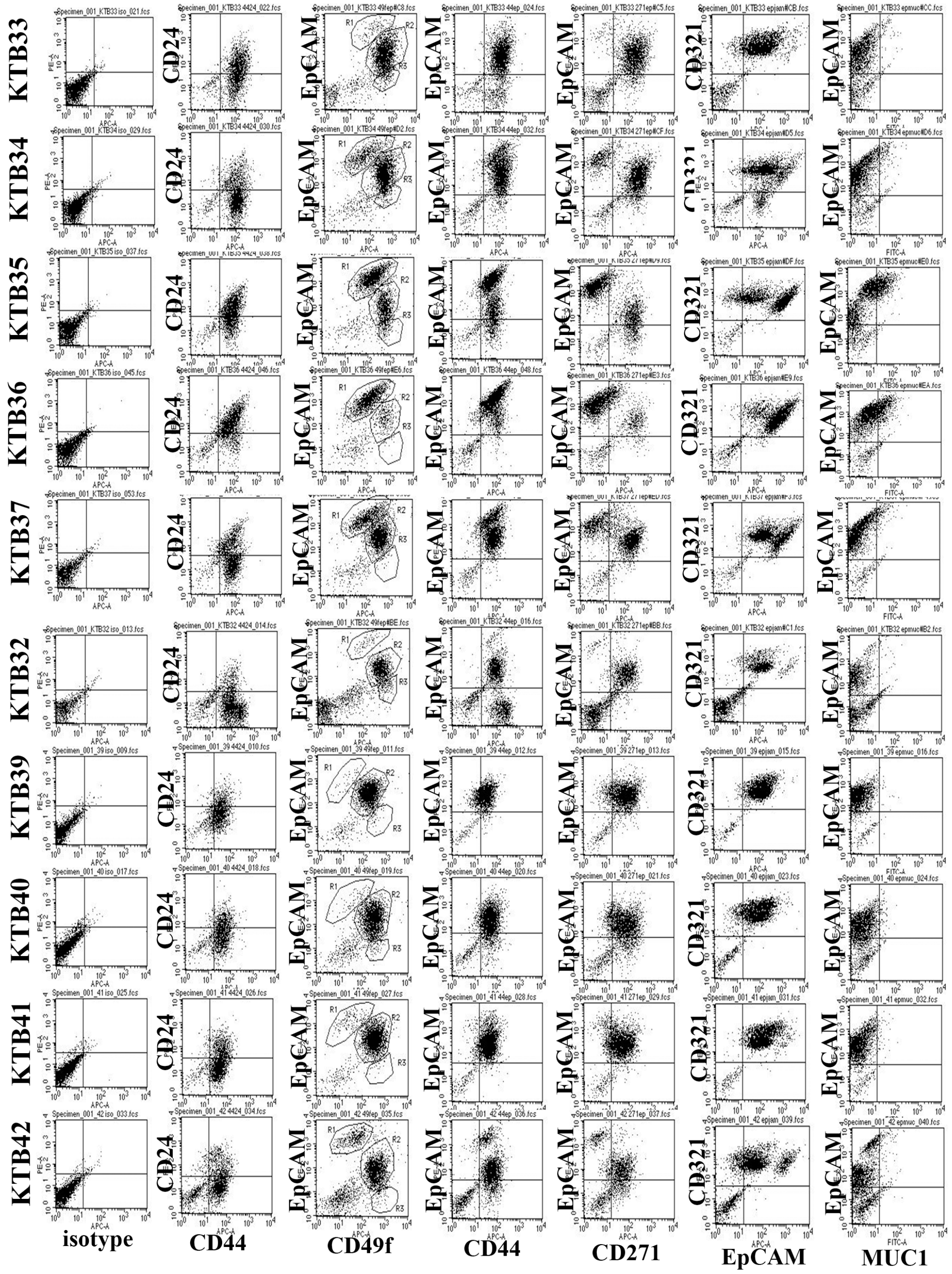

Figure S4: Documenting heterogeneity in breast epithelial cells of healthy donors measured using ALDEFLUOR staining. Cells were stained with ALDEFLUOR. DEAB, a competitive inhibitor of ALDH, was used as a negative control and to establish the baseline fluorescence of these cells.

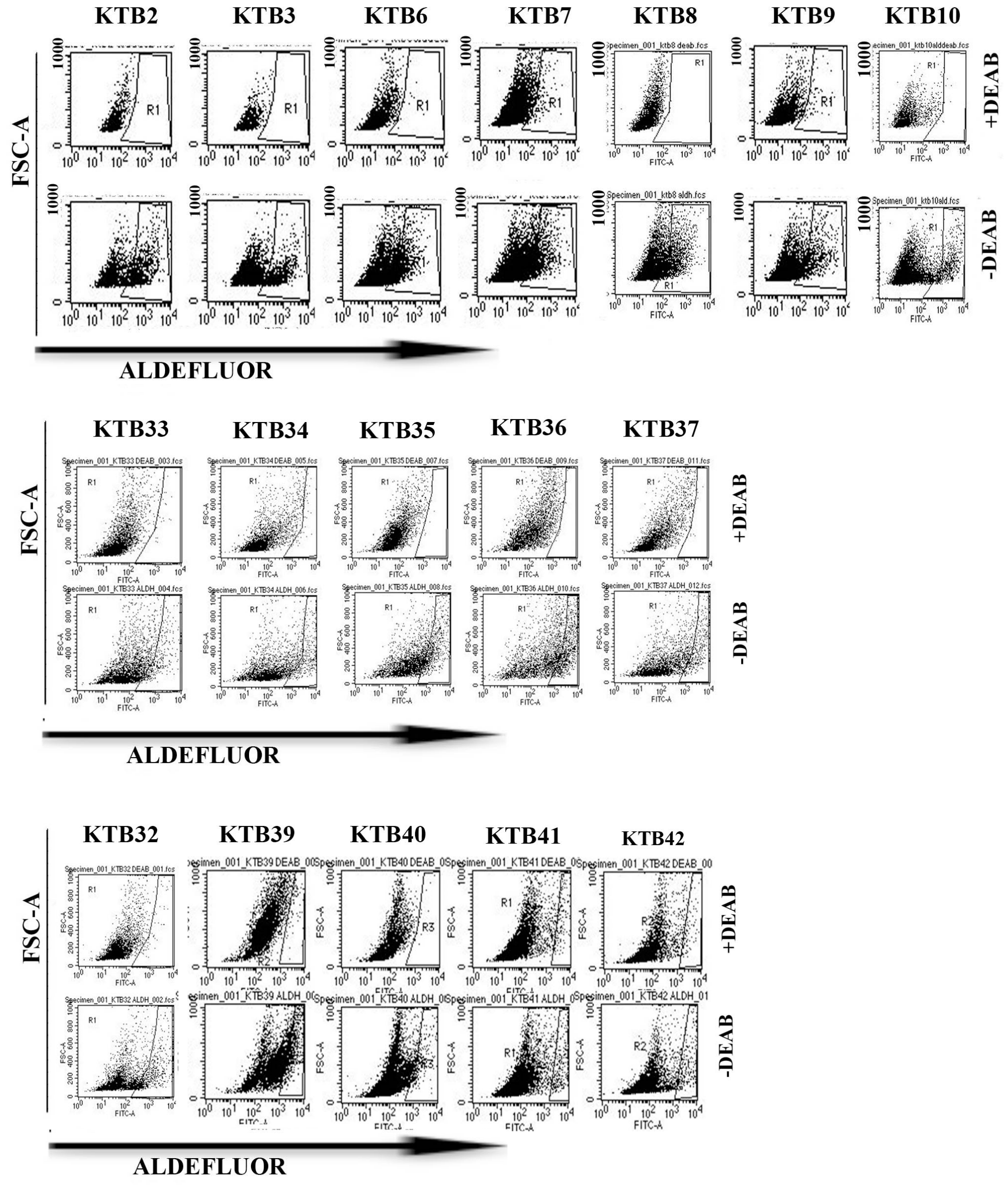

Figure S5: A) Irradiated fibroblasts do not stain for most of the cell surface markers or ALDEFLUOR. B) Epithelial cells grown under reprogramming assay contain <4% contaminating hematopoietic (CD45+) and endothelial cells (CD31+) cells. These cells did not stain for CD140b suggesting lack of fibroblast contamination. CD44/CD24 and CD49f/EpCAM staining patterns were not significantly affected if CD31 and CD45-positive populations were included or excluded in the analyses. C) Pan-cytokeratin antibody staining pattern of epithelial cells.

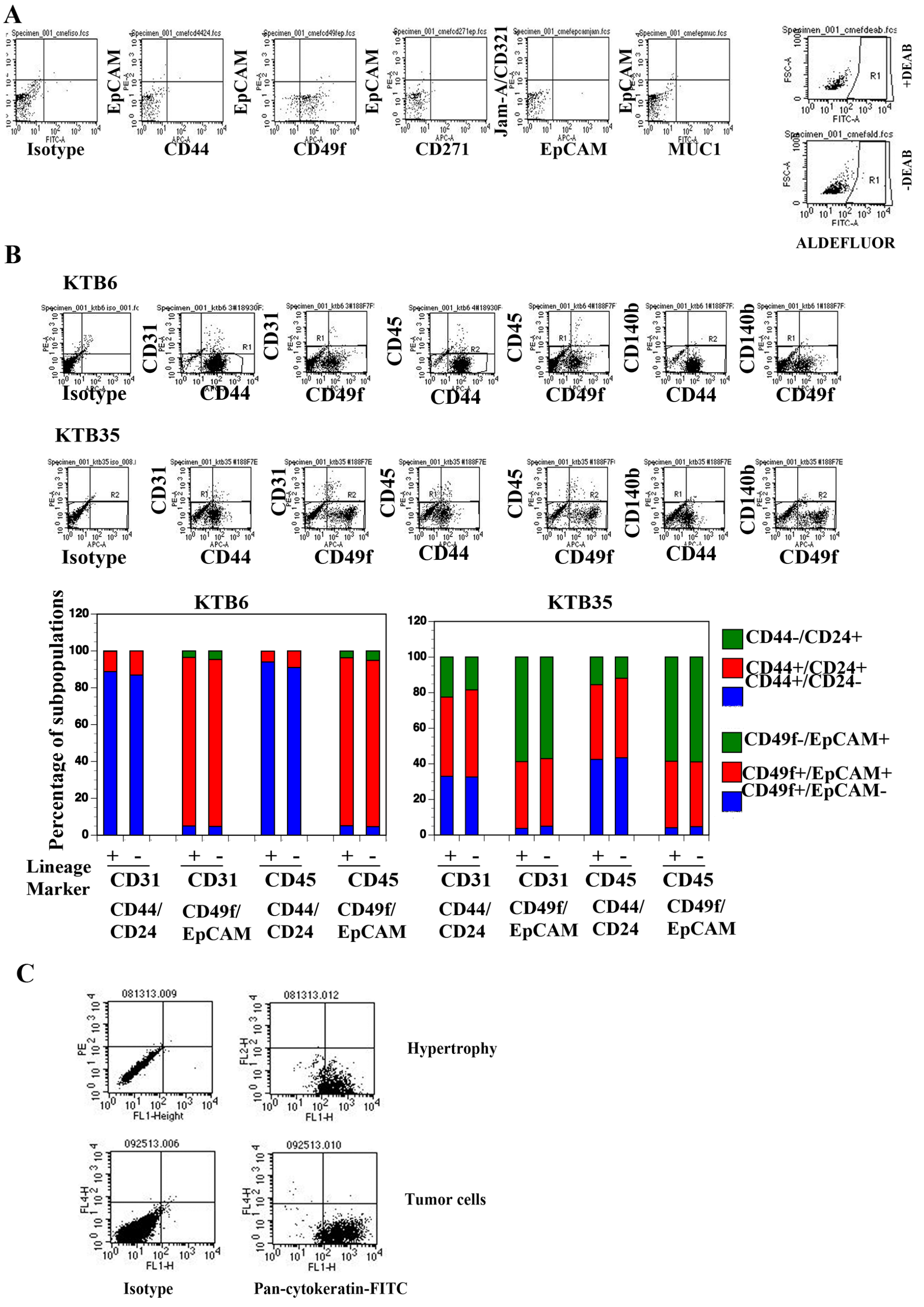

Figure S6: Pathway analysis of genes differentially expressed in CD44^{high}/CD24- subpopulation compared with CD44+/CD24- cells. A) Pathway-associated with up-regulated genes in CD44^{high}/CD24- cells. B) Pathway-associated with down-regulated genes in CD44^{high}/CD24- cells.

A Genes elevated in CD44high/CD24- cells

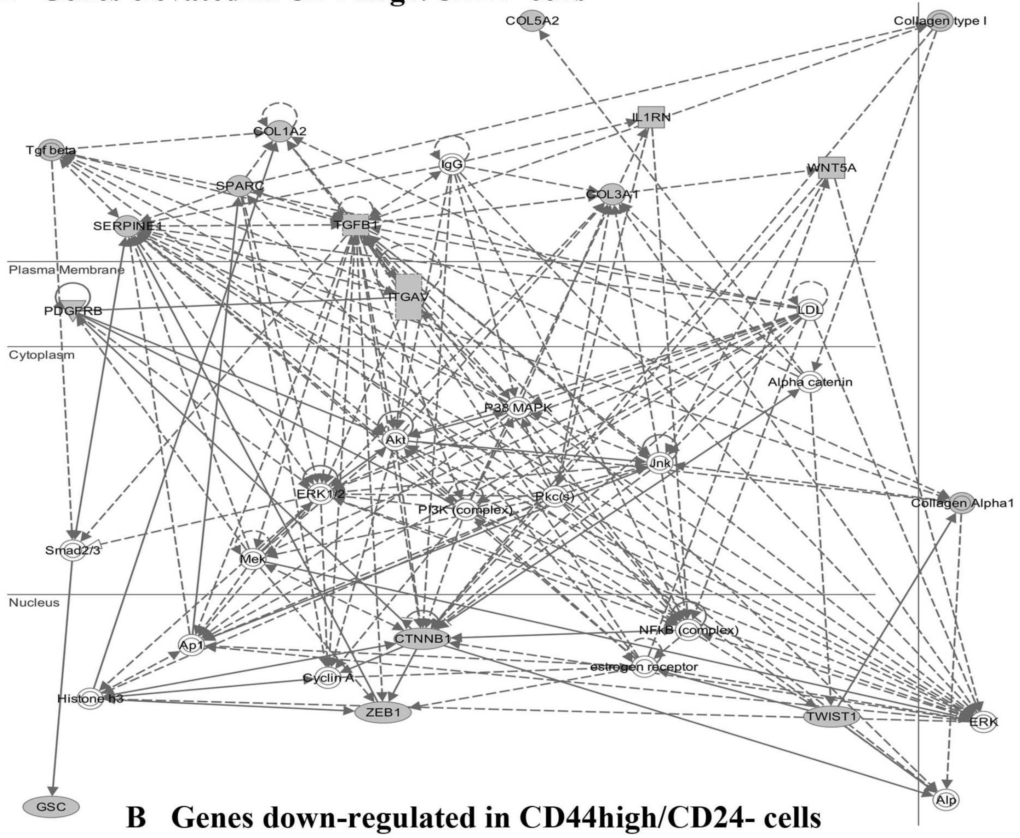

B Genes down-regulated in CD44high/CD24- cells

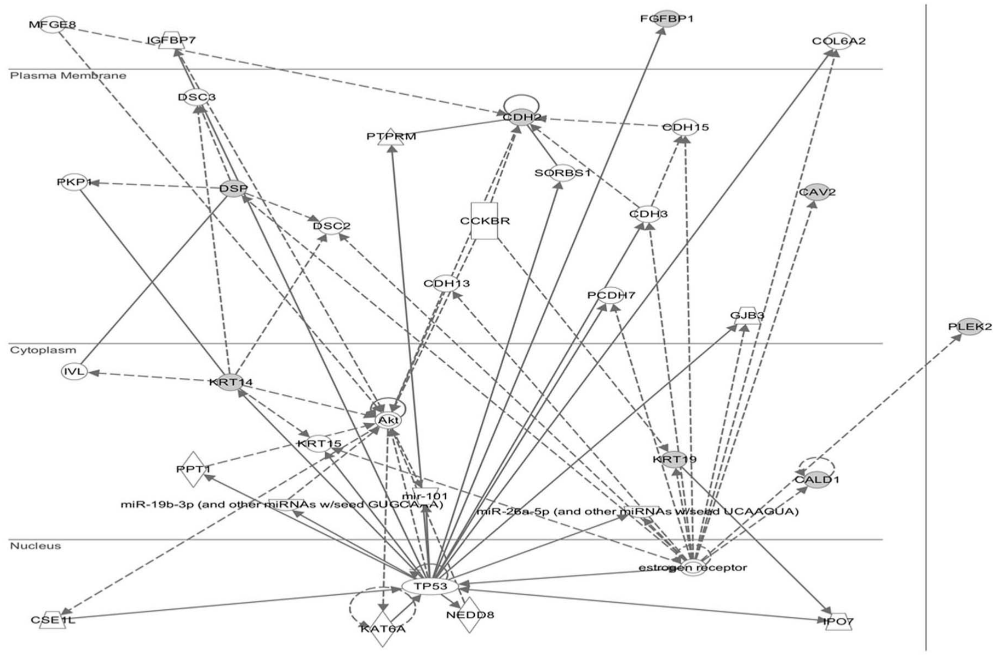

Figure S7: Cells derived from AA women are enriched for PROCR+/EpCAM-cells. Self-reported ethnicity of women is indicated.

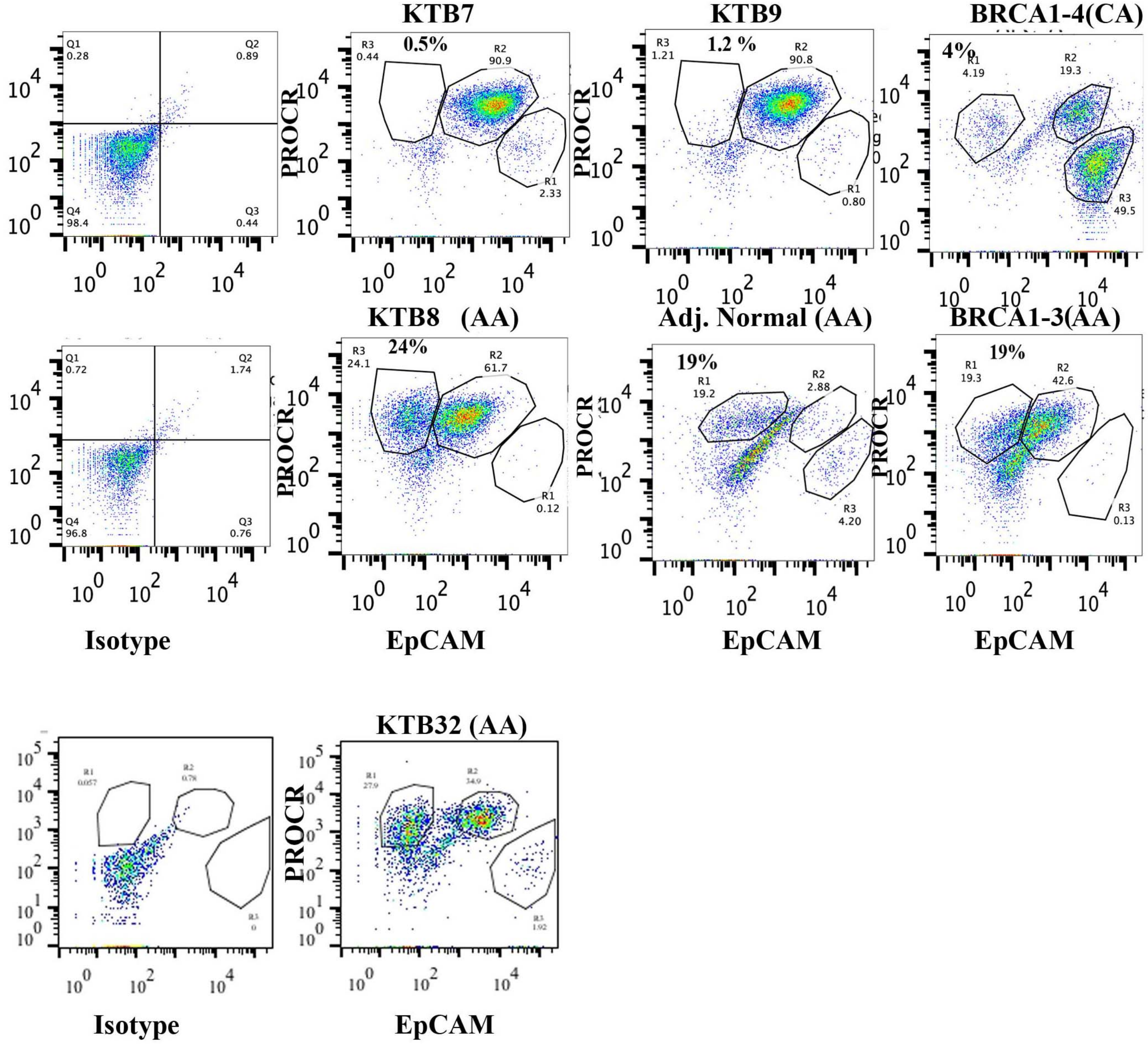

Figure S8: Inter-individual heterogeneity in BRCA1-mutant carriers. A) Breast epithelial cells from four BRCA1-mutant carriers and three BRCA2-mutant carriers were stained with antibodies against the indicated cell surface markers and subjected to flow cytometry. The vertical arrow indicates a unique CD44^{high}/CD24- subpopulation in the AA BRCA1-mutant carrier. B) BRCA1-mutant carriers have lower numbers of ALDEFLUOR+ cells. Assays were performed as in Figure S4.

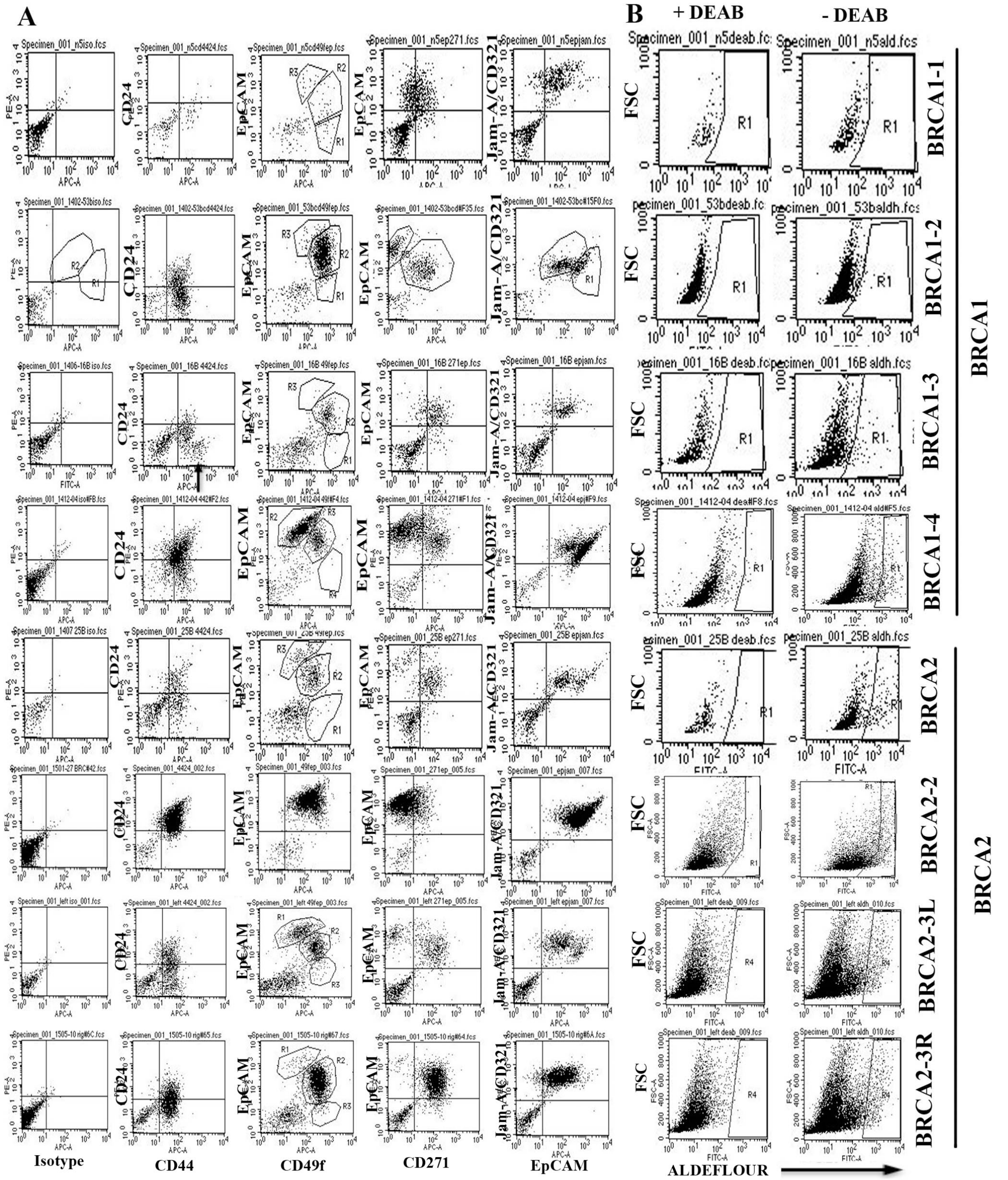

Figure S9: Breast epithelial cells from high-risk patients do not show unique patterns. Breast epithelial cells from a case of hypertrophy, a patient with recurrent breast cancer and a patient with fibrosis were stained with indicated antibody.

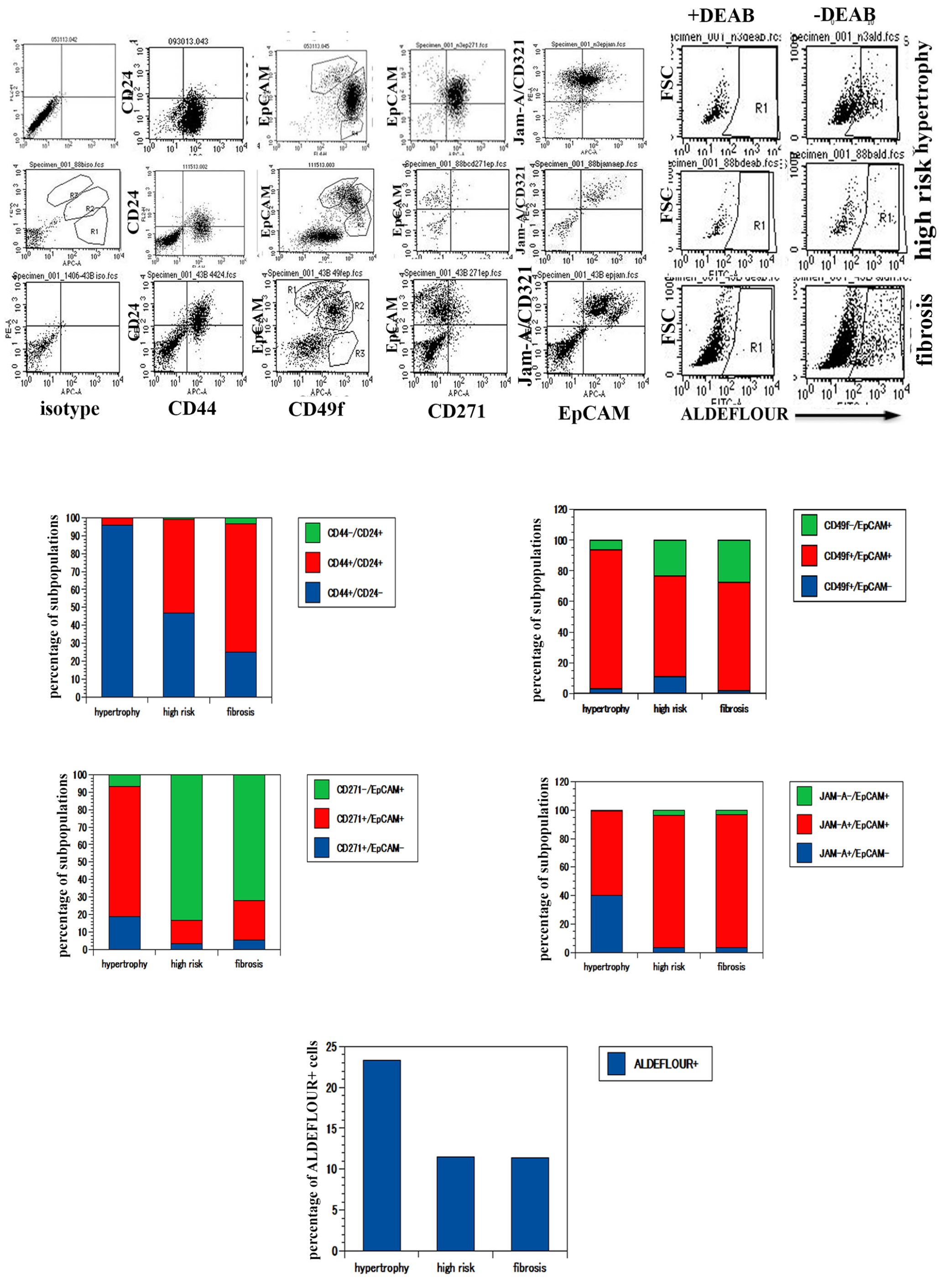

Figure S10: Tumor and adjacent normal cells show differences in CD49f and EpCAM staining pattern. Tumor and adjacent normal cells from six patients were stained with isotype control, CD44-APC/CD24-PE or CD49f-APC/EpCAM-PE and subjected to flow cytometry. The tumor characteristics, age, and ethnicity of the patients are indicated. CD44+/CD24- cells in tumors are suggested to be CSCs. CD49f+/EpCAM-, CD49f+/EpCAM+, and CD49f-/EpCAM+ cells are considered to represent stem, luminal progenitor, and mature/differentiated cells, respectively.

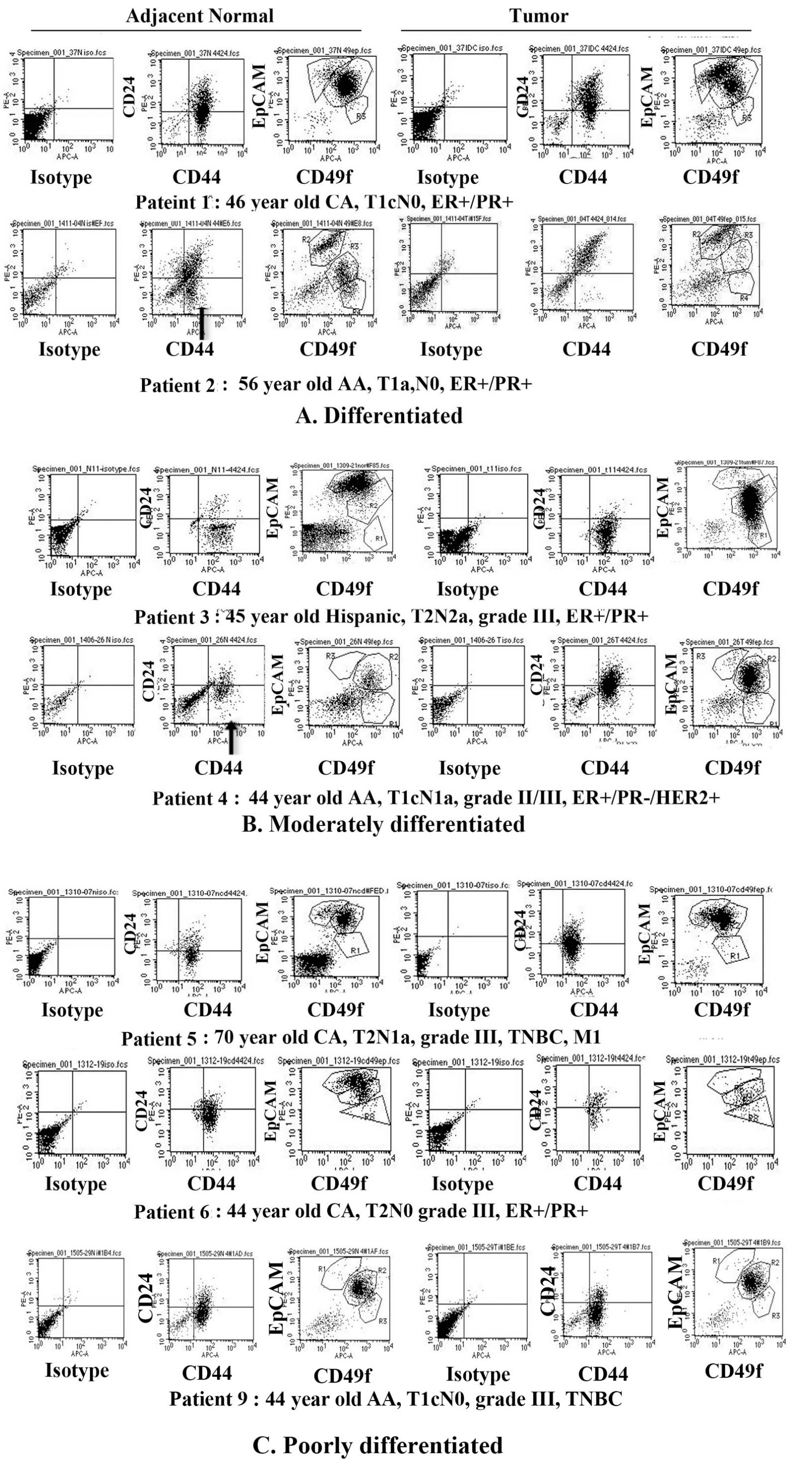

Figure S11: A) CSC-associated marker profile of tumor cells propagated from an ER+/PR+ moderately differentiated tumor. B) CSC-associated marker profile of a TNBC inflammatory breast cancer.

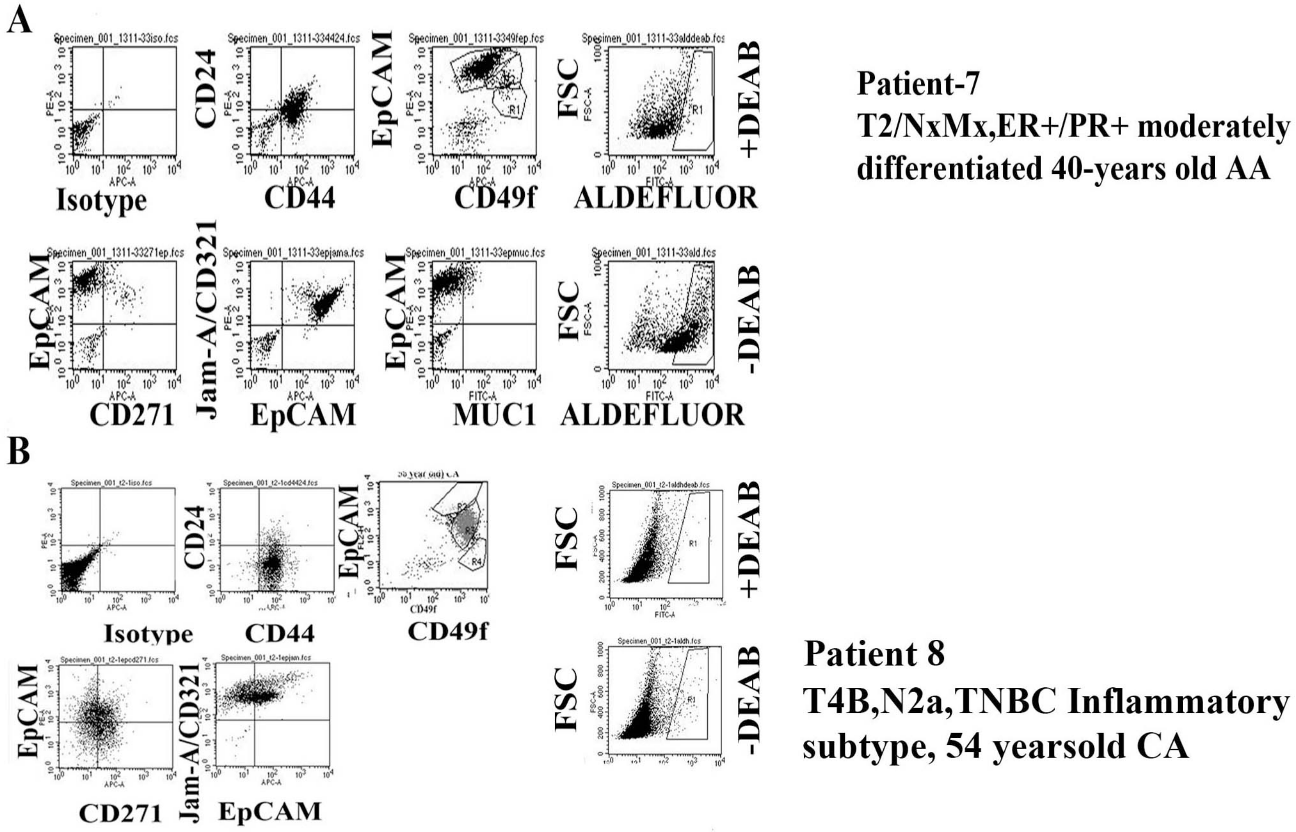

Figure S12: Tumor and adjacent normal cells show differences in ALDEFLUOR+ cells. A) The ALDEFLUOR staining pattern in tumor and adjacent normal cells from six patients. All but two tumors contained higher ALDEFLUOR+ cells compared with adjacent normal cells. B). CD271-APC/EpCAM-PE staining pattern of tumor and adjacent normal cells. C) Jam-A-PE/EpCAM-APC staining pattern of tumor and adjacent normal cells.

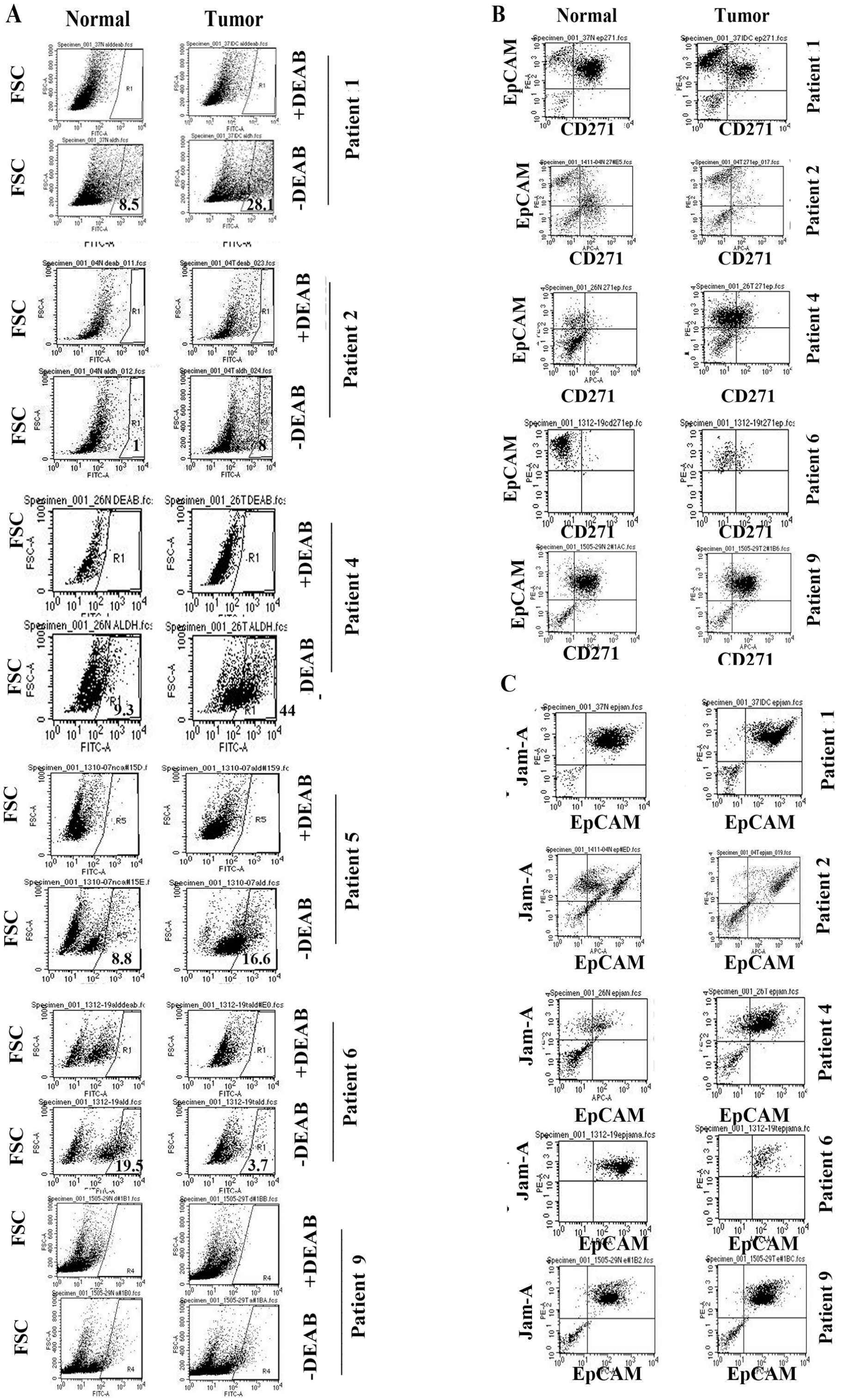

Figure S13: Cell surface markers and ALDEFLUOR staining pattern of cells obtained from two breasts of a woman with benign disease.

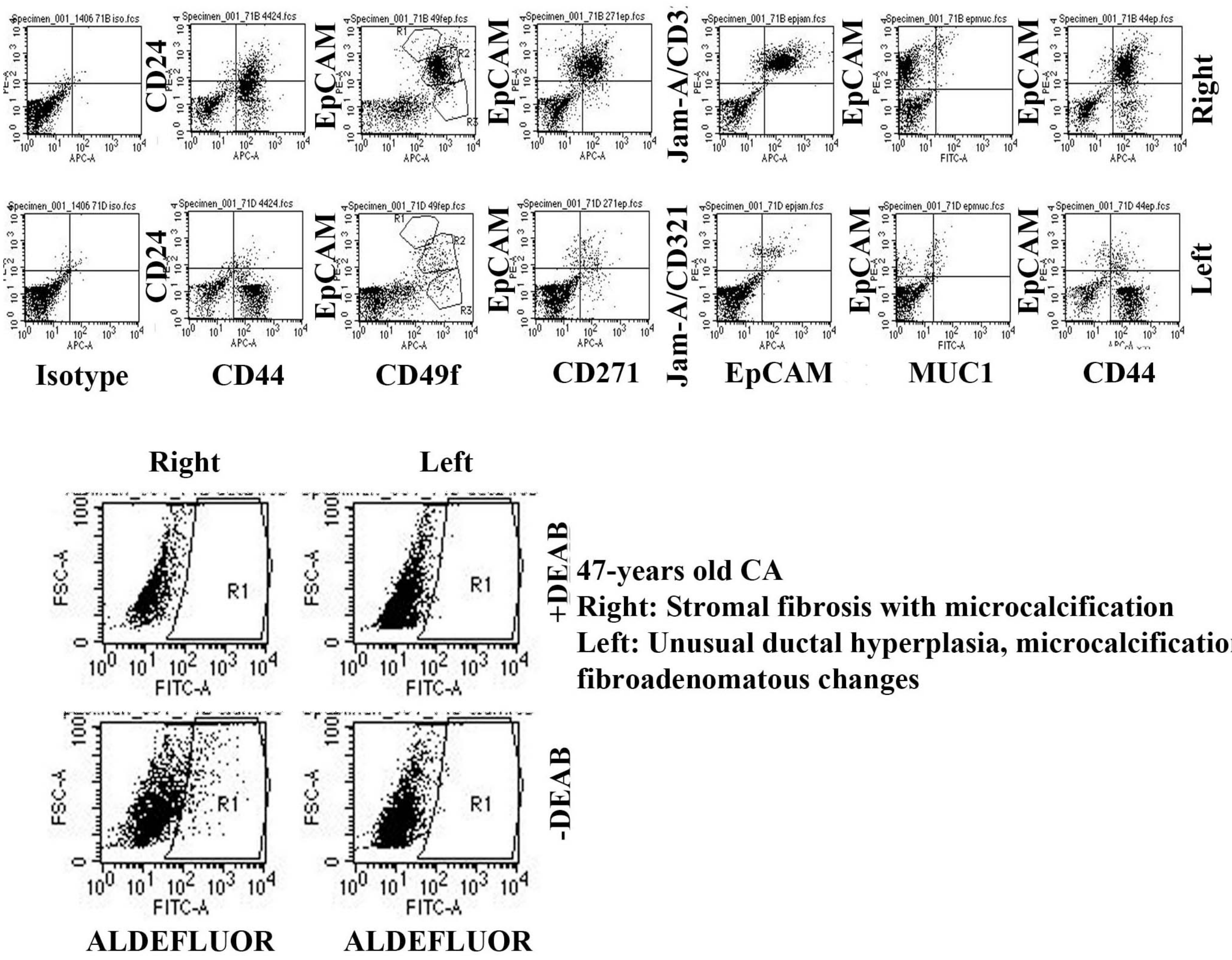

47-years old CA
Right: Stromal fibrosis with microcalcification
Left: Unusual ductal hyperplasia, microcalcification, fibroadenomatous changes
