# Supplementary material for: Ethnicity-Dependent and -Independent Heterogeneity in Healthy Normal Breast Hierarchy Impacts Tumor Characterization
Source: Sci Rep. 2015 Aug 27;5:13526. doi: 10.1038/srep13526 (PMC4550930; doi:10.1038/srep13526)
Supplement: Supplementary Table S2 [file srep13526-s2.pdf]

Ethnicity-Dependent and -Independent Heterogeneity in Healthy Normal Breast Hierarchy Impacts Tumor Characterization

Harikrishna Nakshatri[1,2,3,4*], Manjushree Anjanappa[1], and Poornima Bhat-Nakshatri[1],

Departments of Surgery[1], Biochemistry and Molecular Biology[2], Center for Computational Biology and Bioinformatics[3], Indiana University School of Medicine, Indianapolis, IN 46202, USA; VA Roudebush Medical Center[4], Indianapolis, IN 46202, USA

Supplementary data Table S2

Symbol	Well	AVG ΔC_t (Ct(GOI) - Ave Ct (HKG))		$2^{-\Delta C_t}$		Fold Change	T-TEST	Fold Up- or Down-Regulation	Comments
		KTB8 CD44+	KTB8 CD44+/24+	KTB8 CD44+	KTB8 CD44+/24+	KTB8 CD44+ /KTB8 CD44+/24+	p value	KTB8 CD44+ /KTB8 CD44+/24+	
AHNAK	A01	3.23	3.22	1.1E-01	1.1E-01	1.00	0.912197	-1.00	C
AKT1	A02	3.83	4.02	7.0E-02	6.2E-02	1.14	0.971183	1.14	C
BMP1	A03	3.89	4.99	6.7E-02	3.2E-02	2.13	0.117060	2.13	C
BMP7	A04	9.29	7.85	1.6E-03	4.3E-03	0.37	0.149257	-2.71	C
CALD1	A05	10.03	8.29	9.6E-04	3.2E-03	0.30	0.260638	-3.34	C
CAMK2N1	A06	0.83	2.22	5.6E-01	2.1E-01	2.63	0.041264	2.63	C
CAV2	A07	4.39	2.12	4.8E-02	2.3E-01	0.21	0.017252	-4.83	C
CDH1	A08	3.79	3.52	7.2E-02	8.7E-02	0.83	0.474747	-1.21	C
CDH2	A09	5.06	2.19	3.0E-02	2.2E-01	0.14	0.018398	-7.33	C
COL1A2	A10	4.13	7.15	5.7E-02	7.0E-03	8.15	0.087698	8.15	C
COL3A1	A11	-2.37	1.42	5.2E+00	3.7E-01	13.86	0.041314	13.86	C
COL5A2	A12	-0.97	2.35	2.0E+00	2.0E-01	10.03	0.046034	10.03	C
CTNNB1	B01	0.79	4.15	5.8E-01	5.6E-02	10.27	0.012413	10.27	C
DSC2	B02	4.43	4.42	4.6E-02	4.7E-02	1.00	0.731507	-1.00	C
DSP	B03	6.83	3.92	8.8E-03	6.6E-02	0.13	0.015141	-7.50	C
EGFR	B04	2.13	0.95	2.3E-01	5.2E-01	0.44	0.007340	-2.26	C
ERBB3	B05	4.56	3.32	4.2E-02	1.0E-01	0.42	0.122344	-2.36	C
ESR1	B06	8.39	7.62	3.0E-03	5.1E-03	0.59	0.780092	-1.71	C
F11R	B07	8.09	7.42	3.7E-03	5.8E-03	0.63	0.615789	-1.59	C
FGFBP1	B08	6.36	4.22	1.2E-02	5.4E-02	0.23	0.051791	-4.41	C
FN1	B09	6.33	4.35	1.2E-02	4.9E-02	0.25	0.245602	-3.93	C
FOXC2	B10	-3.34	-1.95	1.0E+01	3.9E+00	2.63	0.035537	2.63	C
FZD7	B11	6.23	6.49	1.3E-02	1.1E-02	1.20	0.885964	1.20	C
GNG11	B12	7.43	7.69	5.8E-03	4.9E-03	1.20	0.976920	1.20	C
GSC	C01	5.26	7.95	2.6E-02	4.0E-03	6.47	0.013126	6.47	C
GSK3B	C02	9.36	8.45	1.5E-03	2.9E-03	0.53	0.287713	-1.87	C
IGFBP4	C03	4.33	3.15	5.0E-02	1.1E-01	0.44	0.031560	-2.26	C

IL1RN	C04	2.19	6.49	2.2E-01	1.1E-02	**19.61**	0.051503	**19.61**	C
ILK	C05	6.86	3.49	8.6E-03	8.9E-02	**0.10**	0.119825	**-10.36**	C
ITGA5	C06	2.89	3.92	1.3E-01	6.6E-02	2.04	0.128279	2.04	C
ITGAV	C07	1.03	3.65	4.9E-01	7.9E-02	**6.18**	**0.024778**	**6.18**	C
ITGB1	C08	3.69	4.19	7.7E-02	5.5E-02	1.41	0.202542	1.41	C
JAG1	C09	0.69	0.35	6.2E-01	7.8E-01	0.79	0.113321	-1.27	C
KRT14	C10	4.46	3.22	4.5E-02	1.1E-01	0.42	**0.009191**	-2.36	C
KRT19	C11	0.93	-2.98	5.3E-01	7.9E+00	**0.07**	**0.025691**	**-15.00**	C
KRT7	C12	8.76	7.15	2.3E-03	7.0E-03	**0.33**	0.237295	**-3.05**	C
MAP1B	D01	2.43	0.02	1.9E-01	9.9E-01	**0.19**	0.229348	**-5.30**	C
MITF	D02	3.13	4.75	1.1E-01	3.7E-02	**3.09**	0.098113	**3.09**	C
MMP2	D03	5.06	5.05	3.0E-02	3.0E-02	1.00	0.763075	-1.00	C
MMP3	D04	8.29	8.55	3.2E-03	2.7E-03	1.20	0.587971	1.20	C
MMP9	D05	7.89	5.55	4.2E-03	2.1E-02	**0.20**	0.064449	**-5.06**	C
MSN	D06	2.09	2.32	2.3E-01	2.0E-01	1.17	0.707753	1.17	C
MST1R	D07	8.59	7.79	2.6E-03	4.5E-03	0.57	0.363787	-1.75	C
NODAL	D08	8.56	8.25	2.6E-03	3.3E-03	0.81	0.945023	-1.24	C
NOTCH1	D09	7.46	6.75	5.7E-03	9.3E-03	0.61	0.269316	-1.63	C
NUDT13	D10	8.36	7.55	3.0E-03	5.3E-03	0.57	0.389204	-1.75	C
OCLN	D11	7.16	5.59	7.0E-03	2.1E-02	0.34	0.061550	-2.98	C
PDGFRB	D12	4.19	7.95	5.5E-02	4.0E-03	**13.55**	**0.017213**	**13.55**	C
PLEK2	E01	7.69	3.95	4.8E-03	6.5E-02	**0.07**	**0.001594**	**-13.36**	C
PPPDE2	E02	3.56	2.89	8.5E-02	1.4E-01	0.63	0.061841	-1.59	C
PTK2	E03	5.73	5.29	1.9E-02	2.6E-02	0.74	0.380554	-1.36	C
PTP4A1	E04	2.53	2.19	1.7E-01	2.2E-01	0.79	0.345203	-1.27	C
RAC1	E05	2.59	2.19	1.7E-01	2.2E-01	0.75	0.353317	-1.33	C
RGS2	E06	7.03	6.22	7.7E-03	1.3E-02	0.57	0.390518	-1.75	C
SERPINE1	E07	-2.27	0.32	4.8E+00	8.0E-01	**6.03**	**0.006009**	**6.03**	C
SIP1	E08	5.69	5.35	1.9E-02	2.4E-02	0.79	0.615192	-1.27	C
SMAD2	E09	4.76	4.89	3.7E-02	3.4E-02	1.09	0.780876	1.09	C
SNAI1	E10	8.93	10.09	2.1E-03	9.2E-04	2.23	0.370510	2.23	C
SNAI2	E11	1.33	1.85	4.0E-01	2.8E-01	1.44	0.426364	1.44	C

SNAI3	E12	9.03	8.42	1.9E-03	2.9E-03	0.66	0.577108	-1.52	C
SOX10	F01	8.53	8.09	2.7E-03	3.7E-03	0.74	0.893294	-1.36	C
SPARC	F02	-1.21	1.69	2.3E+00	3.1E-01	**7.43**	**0.033574**	**7.43**	C
SPP1	F03	8.79	8.29	2.3E-03	3.2E-03	0.70	0.741668	-1.42	C
STAT3	F04	4.39	3.75	4.8E-02	7.4E-02	0.64	0.074908	-1.56	C
STEAP1	F05	5.33	5.49	2.5E-02	2.2E-02	1.12	0.865424	1.12	C
TCF3	F06	5.59	4.55	2.1E-02	4.3E-02	0.49	0.155955	-2.06	C
TCF4	F07	4.89	5.62	3.4E-02	2.0E-02	1.65	0.179796	1.65	C
TFPI2	F08	1.76	1.15	3.0E-01	4.5E-01	0.66	0.479779	-1.52	C
TGFB1	F09	2.89	4.32	1.3E-01	5.0E-02	2.69	**0.008049**	2.69	C
TGFB2	F10	5.99	5.79	1.6E-02	1.8E-02	0.87	0.731903	-1.15	C
TGFB3	F11	7.83	7.75	4.4E-03	4.6E-03	0.95	0.883954	-1.05	C
TIMP1	F12	1.03	3.49	4.9E-01	8.9E-02	**5.50**	0.079558	**5.50**	C
TMEFF1	G01	7.16	7.32	7.0E-03	6.3E-03	1.12	0.885164	1.12	C
TMEM132A	G02	7.43	6.29	5.8E-03	1.3E-02	0.45	0.222740	-2.20	C
TSPAN13	G03	8.86	6.59	2.2E-03	1.0E-02	**0.21**	0.073299	**-4.83**	C
TWIST1	G04	5.23	8.32	2.7E-02	3.1E-03	**8.53**	**0.015837**	**8.53**	C
VCAN	G05	4.29	5.89	5.1E-02	1.7E-02	**3.02**	0.142889	**3.02**	C
VIM	G06	-1.84	-1.05	3.6E+00	2.1E+00	1.73	0.092964	1.73	C
VPS13A	G07	6.29	5.42	1.3E-02	2.3E-02	0.55	0.152321	-1.83	C
WNT11	G08	11.43	9.59	3.6E-04	1.3E-03	**0.28**	0.253330	**-3.58**	C
WNT5A	G09	1.86	4.62	2.8E-01	4.1E-02	**6.77**	**0.011458**	**6.77**	C
WNT5B	G10	4.19	5.12	5.5E-02	2.9E-02	1.90	0.593441	1.90	C
ZEB1	G11	5.46	7.95	2.3E-02	4.0E-03	**5.63**	**0.022017**	**5.63**	C
ZEB2	G12	4.33	6.49	5.0E-02	1.1E-02	**4.47**	0.067988	**4.47**	C
B2M	H01	-3.24	-3.11	9.4E+00	8.7E+00	1.09	0.986231	1.09	C
HPRT1	H02	1.79	2.12	2.9E-01	2.3E-01	1.25	0.445519	1.25	C
RPL13A	H03	-1.61	-1.01	3.0E+00	2.0E+00	1.51	0.400912	1.51	C
GAPDH	H04	4.83	4.42	3.5E-02	4.7E-02	0.75	0.312110	-1.33	C
ACTB	H05	-1.77	-2.41	3.4E+00	5.3E+00	0.64	0.156936	-1.56	C

<u>Legend</u>:

Fold-Change (2^(- Delta Delta Ct)) is the normalized gene expression (2^(- Delta Ct)) in the Test Sample divided the normalized gene expression (2^(- Delta Ct)) in the Control Sample.

Fold-Regulation represents fold-change results in a biologically meaningful way. Fold-change values greater than one indicate a positive- or an up-regulation, and the fold-regulation is equal to the fold-change

Fold-change values less than one indicate a negative or down-regulation, and the fold-regulation is the negative inverse of the fold-change.
Fold-change and fold-regulation values greater than 2 are indicated in red; fold-change values less than 0.5 and fold-regulation values less than -2 are indicated in blue.

p-values: The p values are calculated based on a Student's t-test of the replicate 2^(- Delta Ct) values for each gene in the control group and treatment groups, and p values less than 0.05 are indicated in red.

Comments:

A: This gene's average threshold cycle is relatively high (> 30) in either the control or the test sample, and is reasonably low in the other sample (< 30).

These data mean that the gene's expression is relatively low in one sample and reasonably detected in the other sample suggesting that the actual fold-change value is at least as large as the calculated and reported fold-change result.

This fold-change result may also have greater variations if p value > 0.05; therefore, it is important to have a sufficient number of biological replicates to validate the result for this gene.

B: This gene's average threshold cycle is relatively high (> 30), meaning that its relative expression level is low, in both control and test samples, and the p-value for the fold-change is either unavailable or relatively high (p > 0.05).

This fold-change result may also have greater variations; therefore, it is important to have a sufficient number of biological replicates to validate the result for this gene.
